# Supplementary material for: Persistent telomere cohesion protects aged cells from premature senescence
Source: Nat Commun. 2020 Jul 3;11:3321. doi: 10.1038/s41467-020-17133-4 (PMC7335080; doi:10.1038/s41467-020-17133-4)
Supplement: Supplementary file 1 — Supplementary Information [file 41467_2020_17133_MOESM1_ESM.pdf]

Supplementary Information

Persistent telomere cohesion protects aged cells from premature senescence

Azarm et al.

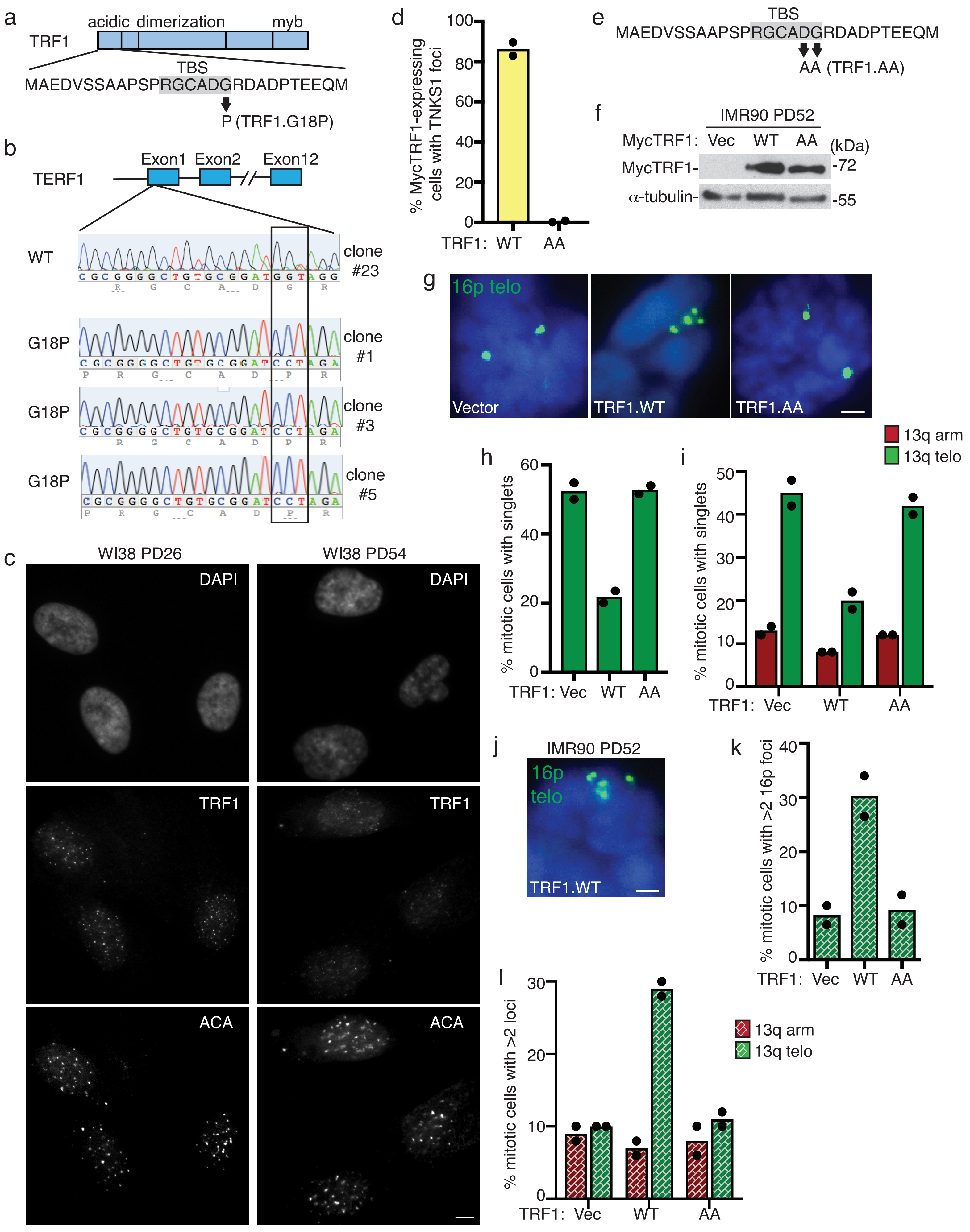

a
TRF1
acidic dimerization myb
TBS
MAEDVSSAAPSPRGCADGRDADPTEEQM
P (TRF1.G18P)
b
TERF1
Exon1 Exon2 Exon12
WT
clone #23
G18P
clone #1
G18P
clone #3
G18P
clone #5
c
WI38 PD26
WI38 PD54
DAPI
TRF1
ACA
d
% MycTRF1-expressing cells with TNKS1 foci
TRF1: WT AA
e
TBS
MAEDVSSAAPSPRGCADGRDADPTEEQM
AA (TRF1.AA)
f
IMR90 PD52
MycTRF1: Vec WT AA
(kDa)
MycTRF1-
-72
α-tubulin-
-55
g
16p telo
Vector
TRF1.WT
TRF1.AA
h
% mitotic cells with singlets
TRF1: Vec WT AA
i
13q arm
13q telo
% mitotic cells with singlets
TRF1: Vec WT AA
j
IMR90 PD52
16p telo
TRF1.WT
k
% mitotic cells with >2 16p foci
TRF1: Vec WT AA
l
% mitotic cells with >2 loci
13q arm
13q telo
TRF1: Vec WT AA

Supplementary Figure 1. Analysis of TRF1.WT versus TRF1.AA in aged primary cells. **a** Schematic diagram highlighting the G18P point mutation generated in HEK293T cells by CRISPR/Cas9 in the tankyrase-binding site (TBS) of TRF1. **b** Schematic diagram and corresponding sequence traces for the tankyrase-binding site of TRF1 in one wild-type HEK293T clone (#23) and three G18P mutant clones (#1, #3, #5). **c** Immunofluorescence analysis of early (PD26) or late (PD54) WI38 cells using anti-TRF1 or anti-centromere (ACA) antibodies. Scale bars represent 5 μm. Images were captured and reproduced under the same conditions and settings. **d** Quantification of the frequency of MycTRF1(WT or AA)-expressing transfected late (PD52) WI38 cells with tankyrase foci. Average of two independent experiments (n≥87 cells each). **e** Schematic diagram highlighting the AA point mutation generated in MycTRF1.WT in the tankyrase-binding site (TBS). **f** Immunoblot analysis of Vector, TRF1.WT, or TRF1.AA transfected late (PD52) IMR90 cell extracts. **g** FISH analysis of Vector, TRF1.WT, or TRF1.AA transfected late (PD52) IMR90 mitotic cells using a 16p telo probe (green). **h** Quantification of the frequency of IMR90 mitotic cells with cohered telomeres. Average of two independent experiments (n≥31 cells each). **i** Quantification of the frequency of mitotic cells with cohered telomeres and arms from FISH analysis of Vector, TRF1.WT, or TRF1.AA transfected late (PD50) WI38 mitotic cells using a dual 13q arm (red) and telo (green) probe. Average of two independent experiments (n=50 cells each). **j** FISH analysis of a TRF1.WT transfected late (PD52) IMR90 mitotic cell exhibiting subtelomere copying using a 16p telo probe (green). **k** Quantification of the frequency of mitotic cells exhibiting subtelomere copying. Average of two independent experiments (n≥31 cells each). **l** Quantification of the frequency of mitotic cells exhibiting subtelomere copying from FISH analysis of Vector, TRF1.WT, or TRF1.AA transfected late (PD50) WI38 mitotic cells using a dual 13q arm (red) and telo (green) probe. Average of two independent experiments (n=50 cells each). (**g, j**) DNA was stained with DAPI (blue). Scale bars represent 2 μm. Experiments were repeated independently twice (for **c, f, g, j**) with similar results. Source data are provided as a Source Data file.

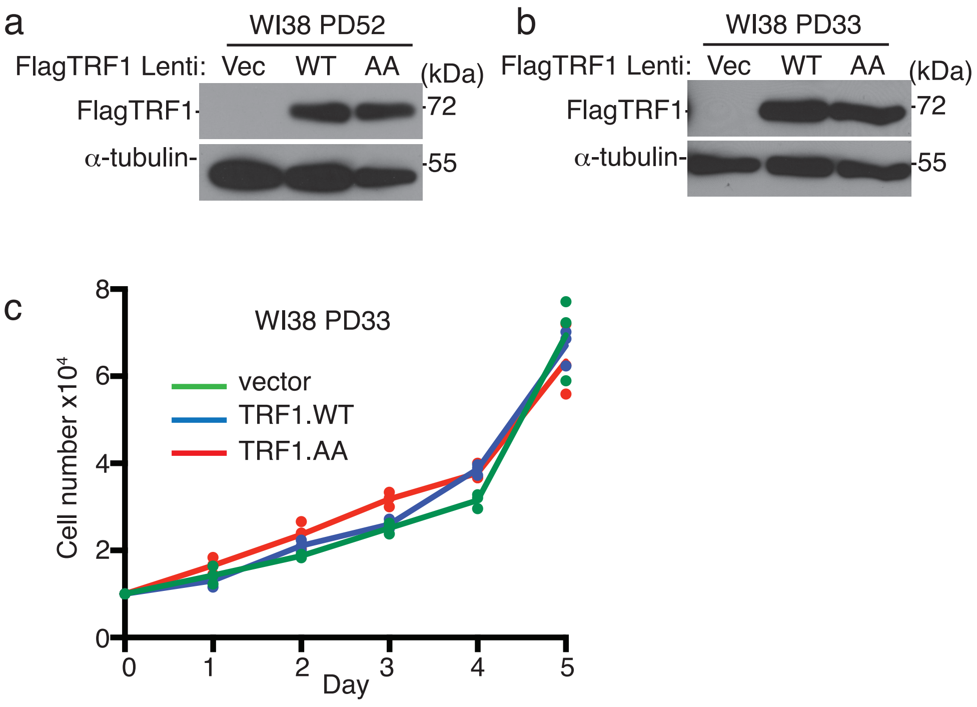

Supplementary Figure 2. TRF1.WT does not affect growth in young WI38 cells.
a, b Immunoblot analysis of Vector, TRF1.WT, or TRF1.AA infected (**a**) late (PD52) or (**b**) early (PD33) WI38 cell extracts. **c** Growth curve analysis of Vector, TRF1.WT, or TRF1.AA infected early (PD33) WI38 cells from three technical replicates. Experiments were repeated independently twice (for **a, b**) with similar results. Source data are provided as a Source Data file.

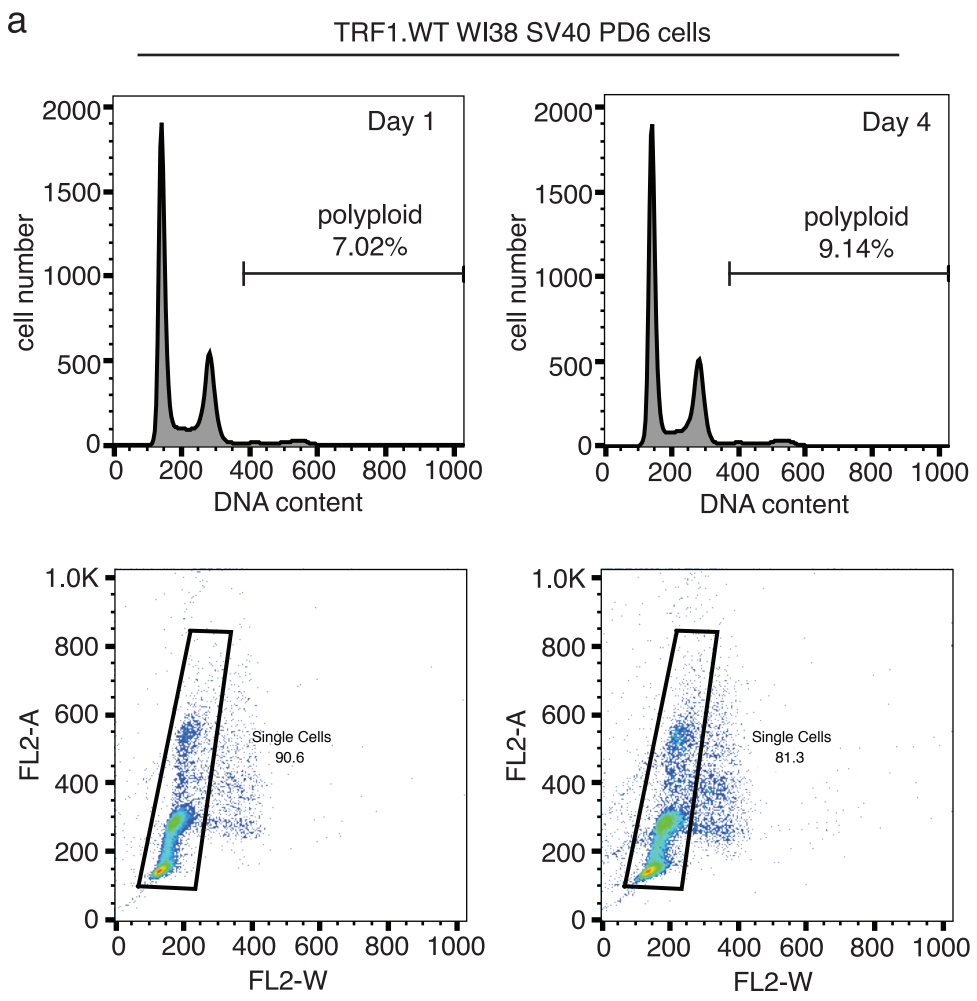

Supplementary Figure 3. TRF1.WT does not affect ploidy in SV40 transformed WI38 cells.
a FACS cell cycle analysis of TRF1.WT infected WI38 SV40-LT (PD6) cells on Day 1 and 4 (n=approximately 20,000 events each). The percentage of events with >4N DNA content (polyploid) is shown. The gating strategy is shown below for each. Single cells were gated (boxed area) and used to generate the histograms.

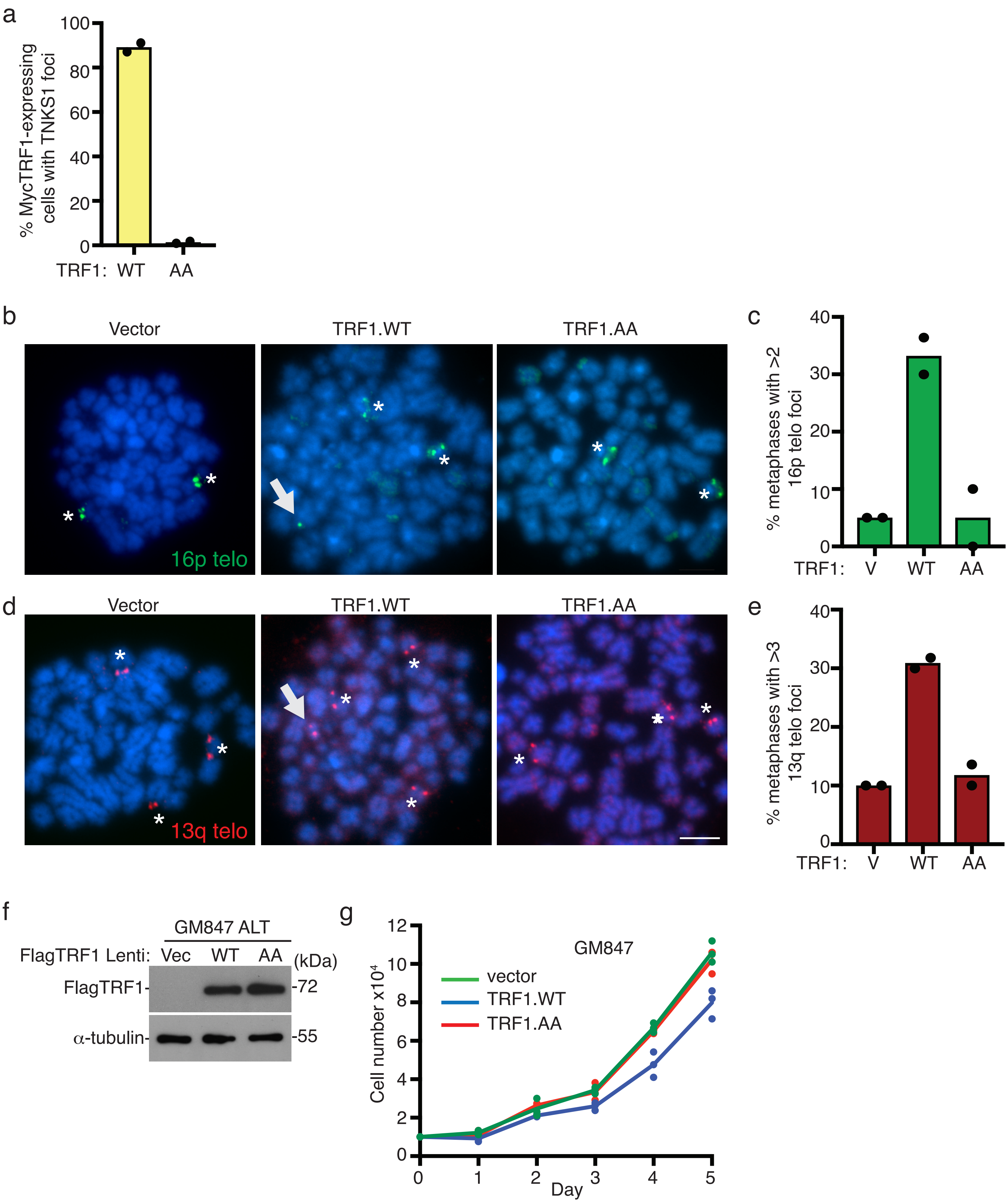
a
% MycTRF1-expressing cells with TNKS1 foci
100
80
60
40
20
0
TRF1: WT AA
b
Vector
TRF1.WT
TRF1.AA
16p telo
c
% metaphases with >2 16p telo foci
40
30
20
10
0
TRF1: V WT AA
d
Vector
TRF1.WT
TRF1.AA
13q telo
e
% metaphases with >3 13q telo foci
40
30
20
10
0
TRF1: V WT AA
f
GM847 ALT
FlagTRF1 Lenti: Vec WT AA
(kDa)
FlagTRF1-
-72
α-tubulin-
-55
g
GM847
vector
TRF1.WT
TRF1.AA
Cell number x10⁴
12
10
8
6
4
2
0
0 1 2 3 4 5
Day

Supplementary Figure 4. Analysis of TRF1.WT versus TRF1.AA in ALT cancer cells.
a Quantification of the frequency of MycTRF1(WT or AA)-expressing transfected GM847 cells with tankyrase foci. Average of two independent experiments (n≥101 cells each). **b, d** FISH analysis of metaphase spreads from Vector, TRF1.WT, or TRF1.AA transfected U2OS cells using (**b**) 16p (green) or (**d**) 13q (triploid in U2OS cells) (red) telo probes. Asterisks indicate usual chromosome number and arrow indicates extra subtelomere copy. **c, e** Quantification of the frequency of metaphases with an extra (**c**) 16p or (**e**) 13q signal. DNA was stained with DAPI (blue). Scale bar represent 5 μm. Average of two independent experiments (n≥20 cells each). **f** Immunoblot analysis of Vector, TRF1.WT, or TRF1.AA infected GM847 cell extracts. **g** Growth curve analysis of Vector, TRF1.WT, or TRF1.AA infected GM847 cells from three technical replicates. (**b, d**) DNA was stained with DAPI (blue). Scale bars represent 2 μm. Experiments were repeated independently twice (for **b, d, f**) with similar results. Source data are provided as a Source Data file.

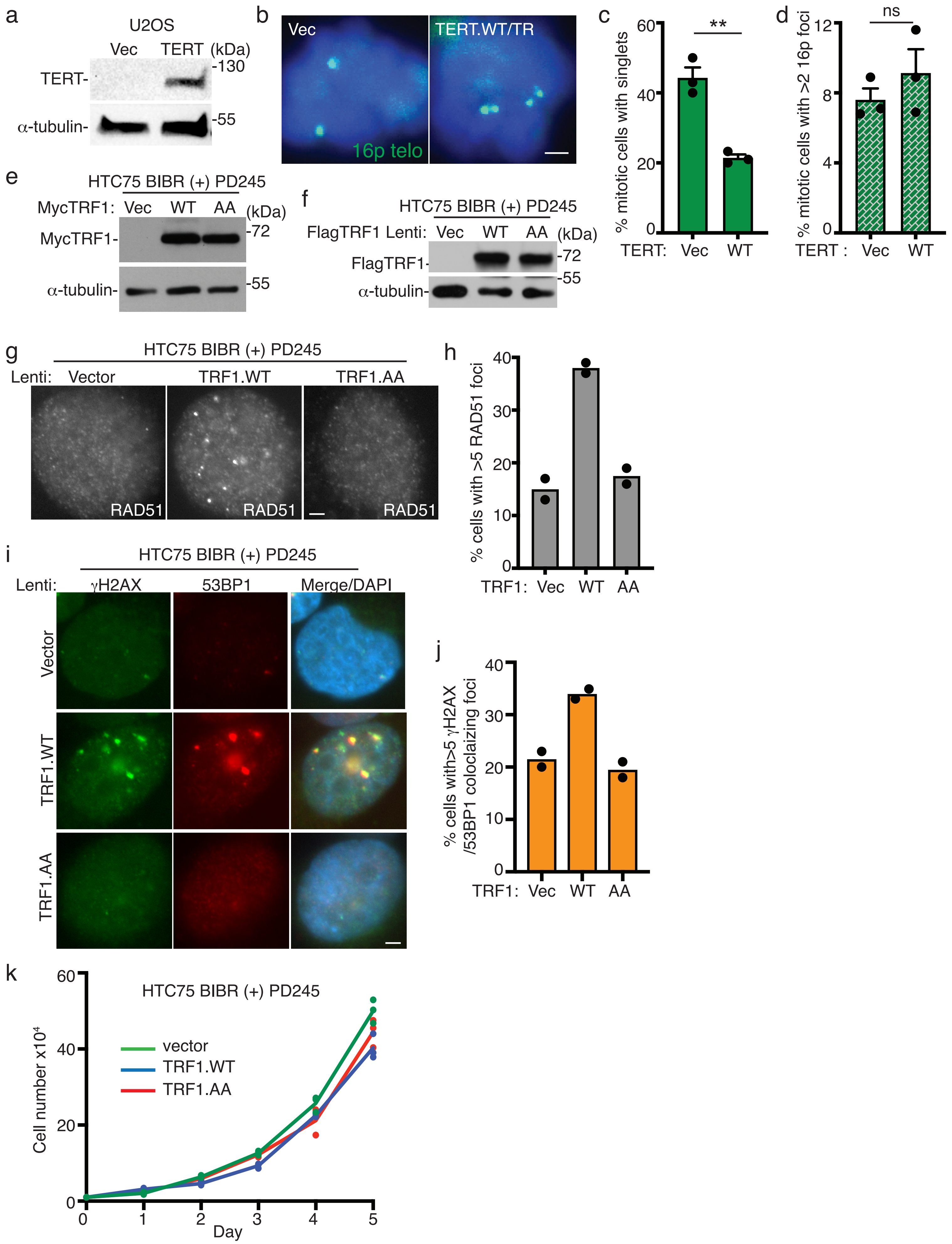

a
U2OS
Vec TERT (kDa)
TERT-
α-tubulin-
-130
-55
b
Vec
TERT.WT/TR
16p telo
c
% mitotic cells with singlets
**
TERT: Vec WT
d
ns
% mitotic cells with >2 16p foci
TERT : Vec WT
e
HTC75 BIBR (+) PD245
MycTRF1: Vec WT AA (kDa)
MycTRF1-
α-tubulin-
-72
-55
f
HTC75 BIBR (+) PD245
FlagTRF1 Lenti: Vec WT AA (kDa)
FlagTRF1-
α-tubulin-
-72
-55
g
HTC75 BIBR (+) PD245
Lenti: Vector TRF1.WT TRF1.AA
RAD51
h
% cells with >5 RAD51 foci
TRF1: Vec WT AA
i
HTC75 BIBR (+) PD245
Lenti: γH2AX 53BP1 Merge/DAPI
Vector
TRF1.WT
TRF1.AA
j
% cells with>5 γH2AX /53BP1 colocalizing foci
TRF1: Vec WT AA
k
HTC75 BIBR (+) PD245
vector
TRF1.WT
TRF1.AA
Cell number x10⁴
Day

Supplementary Figure 5. Consequences of telomerase overexpression or inhibition.
a Immunoblot analysis of Vector and TERT.WT/TR transfected U2OS cell extracts.
b FISH analysis of Vector or TERT.WT/TR transfected U2OS mitotic cells using a 16p telo probe (green). DNA was stained with DAPI (blue). Scale bar represents 2 μm. **c, d** Quantification of the frequency of mitotic cells (**c**) with cohered telomeres or (**d**) exhibiting subtelomere copying. Average of three independent experiments (n≥42 cells each) ± SEM. **c** Vec vs WT: p = 0.0074. **d** Vec vs WT: p = 0.4472. **e** Immunoblot analysis of Vector, TRF1.WT, or TRF1.AA transfected HTC75 BIBR (+) PD245 cell extracts. **f** Immunoblot analysis of Vector, TRF1.WT, or TRF1.AA infected HTC75 BIBR (+) PD245 cell extracts. **g** Immunofluorescence analysis of Vector, TRF1.WT, or TRF1.AA infected HTC75 BIBR (+) PD245 cells with RAD51 antibody. **h** Quantification of the frequency of cells displaying >5 RAD51 foci. Average of two independent experiments (n=100 cells each). **i** Immunofluorescence analysis of Vector, TRF1.WT, or TRF1.AA infected HTC75 BIBR (+) PD245 cells with γH2AX (green) and 53BP1 (red) antibodies. **j** Quantification of the frequency of cells displaying >5 γH2AX/53BP1 colocalizing foci. Average of two independent experiments (n≥100 cells each). **k** Growth curve analysis of Vector, TRF1.WT, or TRF1.AA infected HTC75 BIBR (+) PD245 cells from three technical replicates. (**b, i**) DNA was stained with DAPI (blue). Scale bars represent 2 μm. **$p \leq 0.01$, (ns) not significant, Student's unpaired two-sided t test. Experiments were repeated independently three times (for **a, b,**) and twice (for **e, f, g, i**) with similar results. Source data are provided as a Source Data file.
