# Supplementary material for: Persistent telomere cohesion protects aged cells from premature senescence
Source: Nat Commun. 2020 Jul 3;11:3321. doi: 10.1038/s41467-020-17133-4 (PMC7335080; doi:10.1038/s41467-020-17133-4)
Supplement: Supplementary file 3 — Reporting Summary [file 41467_2020_17133_MOESM3_ESM.pdf]

nature research

Corresponding author(s): Susan Smith

Last updated by author(s): Mar 26, 2020

Reporting Summary

Nature Research wishes to improve the reproducibility of the work that we publish. This form provides structure for consistency and transparency in reporting. For further information on Nature Research policies, see Authors & Referees and the Editorial Policy Checklist .

Statistics

For all statistical analyses, confirm that the following items are present in the figure legend, table legend, main text, or Methods section.

n/a	Confirmed	
[ ]	[x]	The exact sample size (*n*) for each experimental group/condition, given as a discrete number and unit of measurement
[ ]	[x]	A statement on whether measurements were taken from distinct samples or whether the same sample was measured repeatedly
[ ]	[x]	The statistical test(s) used AND whether they are one- or two-sided<br>*Only common tests should be described solely by name; describe more complex techniques in the Methods section.*
[x]	[ ]	A description of all covariates tested
[x]	[ ]	A description of any assumptions or corrections, such as tests of normality and adjustment for multiple comparisons
[ ]	[x]	A full description of the statistical parameters including central tendency (e.g. means) or other basic estimates (e.g. regression coefficient) AND variation (e.g. standard deviation) or associated estimates of uncertainty (e.g. confidence intervals)
[ ]	[x]	For null hypothesis testing, the test statistic (e.g. *F*, *t*, *r*) with confidence intervals, effect sizes, degrees of freedom and *P* value noted<br>*Give P values as exact values whenever suitable.*
[x]	[ ]	For Bayesian analysis, information on the choice of priors and Markov chain Monte Carlo settings
[x]	[ ]	For hierarchical and complex designs, identification of the appropriate level for tests and full reporting of outcomes
[x]	[ ]	Estimates of effect sizes (e.g. Cohen's *d*, Pearson's *r*), indicating how they were calculated

Our web collection on statistics for biologists contains articles on many of the points above.

Software and code

Policy information about availability of computer code

Data collection	For fluorescence microscopy, images were acquired using Openlab 5.5 software (Perkin Elmer).<br>SA-Beta Gal staining was imaged with simple brightfield at 20X magnification using a Zeiss AxioObserver.Z1 microscope.
Data analysis	Statistical analysis was performed using Prism 8 software. FACs analysis was performed using a Becton, Dickinson FACSCalibur and CellQuest Pro software (v5.1), and modeled using FlowJo v9 software.

For manuscripts utilizing custom algorithms or software that are central to the research but not yet described in published literature, software must be made available to editors/reviewers. We strongly encourage code deposition in a community repository (e.g. GitHub). See the Nature Research guidelines for submitting code & software for further information.

Data

Policy information about availability of data

All manuscripts must include a data availability statement. This statement should provide the following information, where applicable:

- Accession codes, unique identifiers, or web links for publicly available datasets
- A list of figures that have associated raw data
- A description of any restrictions on data availability

The authors declare that the data supporting the findings of this study are available within the paper and its supplementary information files. The Source Data underlying the following Figures: 1B, D, E, H, J; 2A-E, G, H; 3B, D, F, H, I, K, M; 4A, C-E, G, H, J: 5A, D, F-J, L, M,); 6A, C, D, G, H, K, L, N, P; and 7B, C, E, G, H, J and Supplementary Figures: S1D, F, H, I, K, L; S2A-C; S4A, C, E, F, G; and S5A, C- F, H, J, K are provided in the Source Data File. All other relevant data are available upon request.

Field-specific reporting

Please select the one below that is the best fit for your research. If you are not sure, read the appropriate sections before making your selection.

☒ Life sciences ☐ Behavioural & social sciences ☐ Ecological, evolutionary & environmental sciences

For a reference copy of the document with all sections, see nature.com/documents/nr-reporting-summary-flat.pdf

Life sciences study design

All studies must disclose on these points even when the disclosure is negative.

Sample size	No sample-size calculation was performed. Sample sizes were based upon experience and similar published studies.<br><br>For number of cells analyzed for FISH, immunofluorescence, and SA-Beta Gal staining, see:<br><br>Daniloski, Z. & Smith, S. Loss of Tumor Suppressor STAG2 Promotes Telomere Recombination and Extends the Replicative Lifespan of Normal Human Cells. Cancer Res 77, 5530-5542 (2017).<br><br>Kim, M.K. & Smith, S. Persistent telomere cohesion triggers a prolonged anaphase. Mol Biol Cell 25, 30-40 (2014).<br><br>For number of cells selected for growth curve seeding, see:<br><br>Ramamoorthy, M. & Smith, S. Loss of ATRX Suppresses Resolution of Telomere Cohesion to Control Recombination in ALT Cancer Cells. Cancer Cell 28, 357-369 (2015).
Data exclusions	No data were excluded from the analysis.
Replication	The number of replications for each experiment are indicated. All attempts at replication were successful.
Randomization	Experiments were performed on non-clonal cell lines; no randomization was performed.
Blinding	Blinding was not done. All experiments were repeated at least twice to ensure reproducibility.

Reporting for specific materials, systems and methods

We require information from authors about some types of materials, experimental systems and methods used in many studies. Here, indicate whether each material, system or method listed is relevant to your study. If you are not sure if a list item applies to your research, read the appropriate section before selecting a response.

Materials & experimental systems

n/a	Involved in the study	
☐	☒	Antibodies
☐	☒	Eukaryotic cell lines
☒	☐	Palaeontology
☒	☐	Animals and other organisms
☒	☐	Human research participants
☒	☐	Clinical data

Methods

n/a	Involved in the study	
☒	☐	ChIP-seq
☐	☒	Flow cytometry
☒	☐	MRI-based neuroimaging

Antibodies

Antibodies used	For immunoblot analysis - rabbit anti-Myc (0.1 µg/ml; Santa Cruz #sc-789), mouse anti-Flag (3.8 µg/ml, Sigma #F3165), rabbit anti-TERT 375 (1:500 dilution of crude serum, raised against Escherichia coli-derived fusion protein containing hTERT amino acids 702-841), mouse anti-POLD3 (2 ug/mL, Abnova #H00010714-M01), or mouse anti-alpha-tubulin ascites (1:10000-1:20000, Sigma #T5168), followed by horseradish peroxidase-conjugated donkey anti-rabbit or sheep anti-mouse IgG (1:3000, Amersham #NA934 or #NA931).<br><br>For immunofluorescence - mouse anti-Myc clone 4A6 (1.0 µg/ml; Milipore #05-724), rabbit anti-tankyrase 1 762 (1.4 µg/ml), mouse anti-gH2AX clone JBW301 (1 µg/ml; Millipore #05-636), rabbit anti-53BP1 (4 µg/ml; Novus Biologicals #NB100-304), rabbit anti-TIN2 701 (0.36 µg/ml), rabbit anti-TRF1 415 (0.2 µg/ml), human anti-centromere (ACA) (1:4000), rabbit anti-RAD51 (4 µg/mL; Santa Cruz #sc-8349), mouse anti-TRF2 (2.5 µg/ml, Imgenex #IMG-124A), followed by FITC-conjugated or TRITC-conjugated donkey anti-rabbit or anti-mouse antibodies (1:100; Jackson Laboratories #711-025-152 or #711-095-152).

Validation

Primary antibodies used for immunoblot analysis in human cells:

rabbit anti-Myc (0.1 µg/ml; Santa Cruz #sc-789) – antibody against myc tag from manufacturer, validated by “Western blot analysis of whole cell lysates prepared from COS cells transfected with a c-Myc fusion protein.”

mouse anti-Flag clone M2 (3.8 µg/ml, Sigma #F3165) – antibody against FLAG tag from manufacturer, initial purification and validation in ‘Brizzard, B.L., et al. Immunoaffinity purification of FLAG epitope-tagged bacterial alkaline phosphatase using a novel monoclonal antibody and peptide elution. BioTechniques, 16, 730-735 (1994).’

rabbit anti-TERT 375 (1:500 dilution of crude serum) – antibody raised against Escherichia coli-derived fusion protein containing hTERT amino acids 702-841. Validated by expected protein size (127 kDa) in transfected cells on Western blot in Fig. 6A and S5A.

mouse anti-POLD3 (2 ug/mL, Abnova #H00010714-M01) – antibody raised against amino acids 357-466 of human POLD3. Validated in previous studies (see ‘Dilley, R.L. et al. Break-induced telomere synthesis underlies alternative telomere maintenance. Nature 539, 54-58 (2016).’), also confirmed by signal loss with siPOLD3 in Fig. 2C and 5G.

mouse anti-alpha-tubulin ascites, clone DM1A (1:10000-1:20000, Sigma #T5168) - antibody against tubulin from manufacturer “derived from the B-5-1-2 hybridoma produced by the fusion of mouse myeloma cells and splenocytes from an immunized mouse.” Validated by manufacturer using Western blot analysis in HeLa cells.

Primary antibodies used for immunofluorescence analysis in human cells:

mouse anti-Myc clone 4A6 (1.0 µg/ml; Milipore #05-724) - antibody against myc tag from manufacturer validated by indirect immunofluorescence of “HeLa cells with or without myc-tagged Scc1.”

rabbit anti-tankyrase 1 762 (1.4 µg/ml) – antibody raised against amino acids 973-1140 of human tankyrase 1, validated in “Scherthan, H. et al. Mammalian meiotic telomeres: protein composition and redistribution in relation to nuclear pores. Mol Biol Cell 11, 4189-4203 (2000).”

mouse anti-gH2AX clone JBW301 (1 µg/ml; Millipore #05-636) – antibody from manufacturer raised against amino acids 134-142 of gamma H2AX, recognizes phosphorylated serine 139. Validated by indirect immunofluorescence of etoposide-treated Jurkat cells.

rabbit anti-53BP1 (4 µg/ml; Novus Biologicals #NB100-304) – antibody from manufacturer raised against amino acids 350-400 of 53BP1. Validated by indirect immunofluorescence of HeLa cells: “53BP1 was detected in immersion fixed HeLa cells (left) but was not detected in 53BP1 knockout Hela cells (right) using Rabbit Anti-human 53BP1 polyclonal antibody (Catalog #NB100-304) at 0.3 ug/mL.”

rabbit anti-TIN2 701 (0.36 µg/ml) – antibody raised against a GST-TIN2C E. coli- derived fusion protein containing N-terminal GST and TIN2 amino-acids 180-254. Validated in ‘Houghtaling, B.R., Cuttonaro, L., Chang, W. & Smith, S. A Dynamic Molecular Link between the Telomere Length Regulator TRF1 and the Chromosome End Protector TRF2. Curr Biol 14, 1621-1631 (2004).’

rabbit anti-TRF1 415 (0.2 µg/ml) – antibody raised against full-length baculovirus-derived TRF1. Validated in ‘Cook, B.D., Dynek, J.N., Chang, W., Shostak, G. & Smith, S. Role for the related poly(ADP-Ribose) polymerases tankyrase 1 and 2 at human telomeres. Mol Cell Biol 22, 332-342. (2002).’

human anti-centromere (ACA) (1:4000) – antibody detecting human centromere, validated in ‘Kim, M.K. & Smith, S. Persistent telomere cohesion triggers a prolonged anaphase. Mol Biol Cell 25, 30-40 (2014).’

rabbit anti-RAD51 (4 µg/mL; Santa Cruz #sc-8349) - antibody from manufacturer raised against amino acids 1-92 of Rad51, validated by indirect immunofluorescence in Hep G2 cells.

mouse anti-TRF2 (2.5 µg/ml, Imgenex #IMG-124A) - antibody from manufacturer, validated by indirect immunofluorescence in U2OS cells.

Eukaryotic cell lines

Policy information about cell lines

Cell line source(s)

HEK293T, WI38, IMR90, Phoenix Amphotrophic, U2OS and GM847 cell lines were obtained from ATCC. 293FT cell lines were obtained from Invitrogen. HTC75 cell lines were obtained from Dr. Titia de Lange.

Authentication

The cell lines were not authenticated.

Mycoplasma contamination

The cell lines were not tested for mycoplasma contamination.

Commonly misidentified lines (See ICLAC register)	None used.

Flow Cytometry

Plots

Confirm that:

- [x] The axis labels state the marker and fluorochrome used (e.g. CD4-FITC).
- [x] The axis scales are clearly visible. Include numbers along axes only for bottom left plot of group (a 'group' is an analysis of identical markers).
- [x] All plots are contour plots with outliers or pseudocolor plots.
- [x] A numerical value for number of cells or percentage (with statistics) is provided.

Methodology

Sample preparation	Following trypsinization at approximately 70% confluence, cells were resuspended in PBS containing 2 mM EDTA, fixed with cold 70% (vol/vol) ethanol, treated with RNAse A (ThermoFisher, 200 μg/ml), stained with propidium iodide (50 μg/ml).
Instrument	Becton, Dickinson FACSCalibur
Software	FlowJo v9
Cell population abundance	At least 20,000 events were modeled for each condition.
Gating strategy	Events were plotted as FL2-A/FL2-W. The same gating was used for both conditions, eliminating doublets.

- [x] Tick this box to confirm that a figure exemplifying the gating strategy is provided in the Supplementary Information.
