# Supplementary material for: Persistent telomere cohesion protects aged cells from premature senescence
Source: Nat Commun. 2020 Jul 3;11:3321. doi: 10.1038/s41467-020-17133-4 (PMC7335080; doi:10.1038/s41467-020-17133-4)

Figure 1E

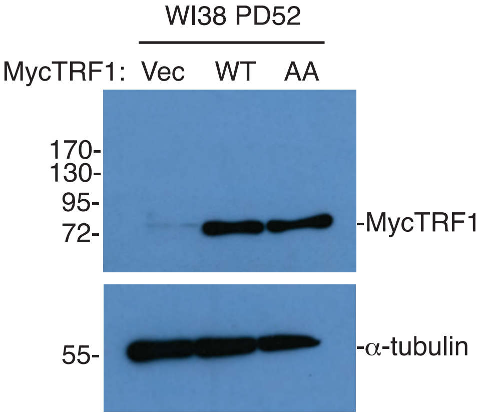

Figure 2C

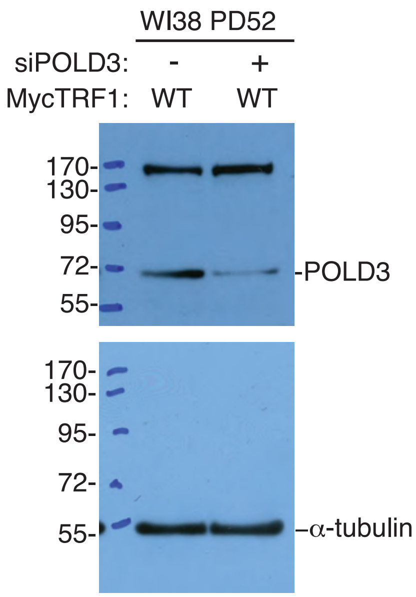

WI38 PD52
siPOLD3: - +
MycTRF1: WT WT
170-
130-
95-
72-
55-
-POLD3
170-
130-
95-
72-
55-
–α-tubulin

Figure 4E

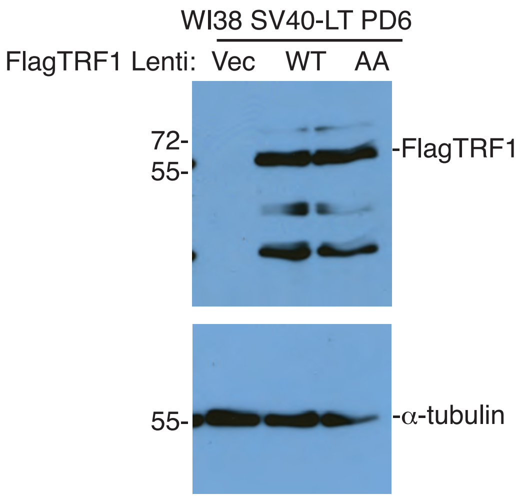

WI38 SV40-LT PD6
FlagTRF1 Lenti: Vec WT AA
72-
55-
-FlagTRF1
55-
-α-tubulin

Figure 5A

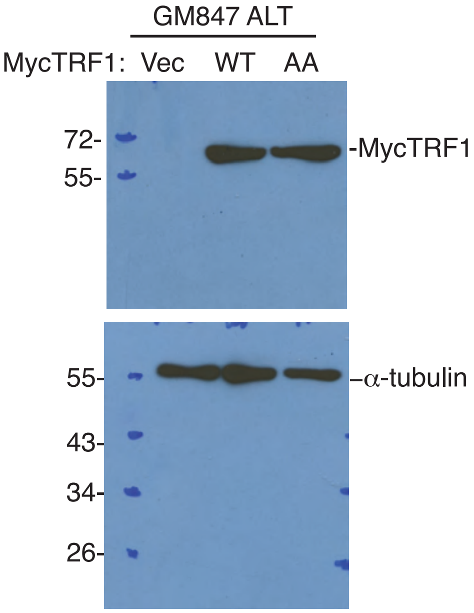

Figure 5G

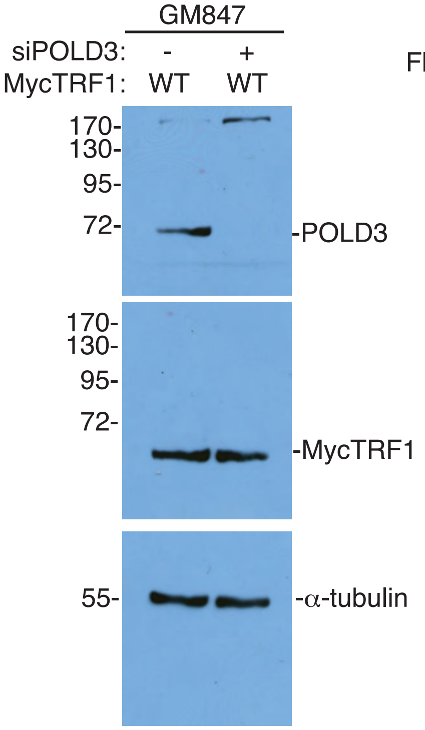

Figure 5J

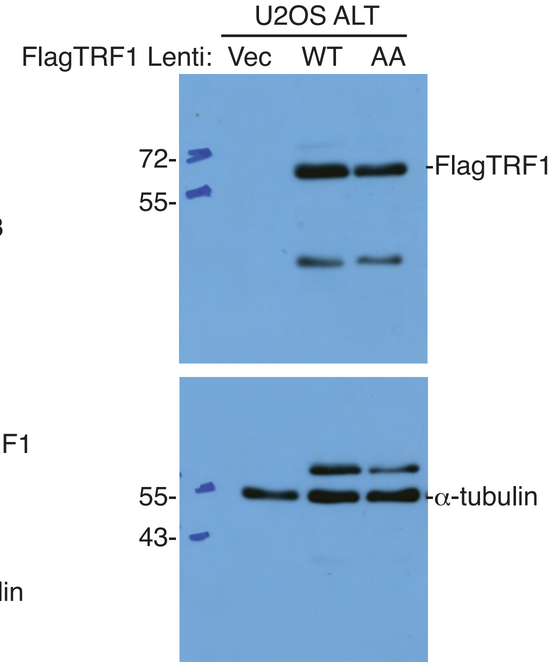

Figure 6A

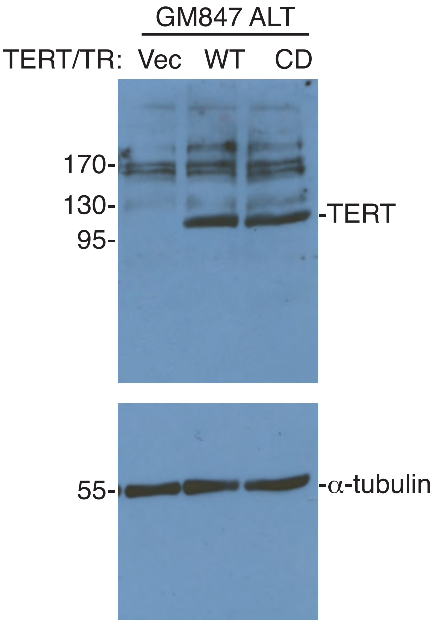

Figure 6H

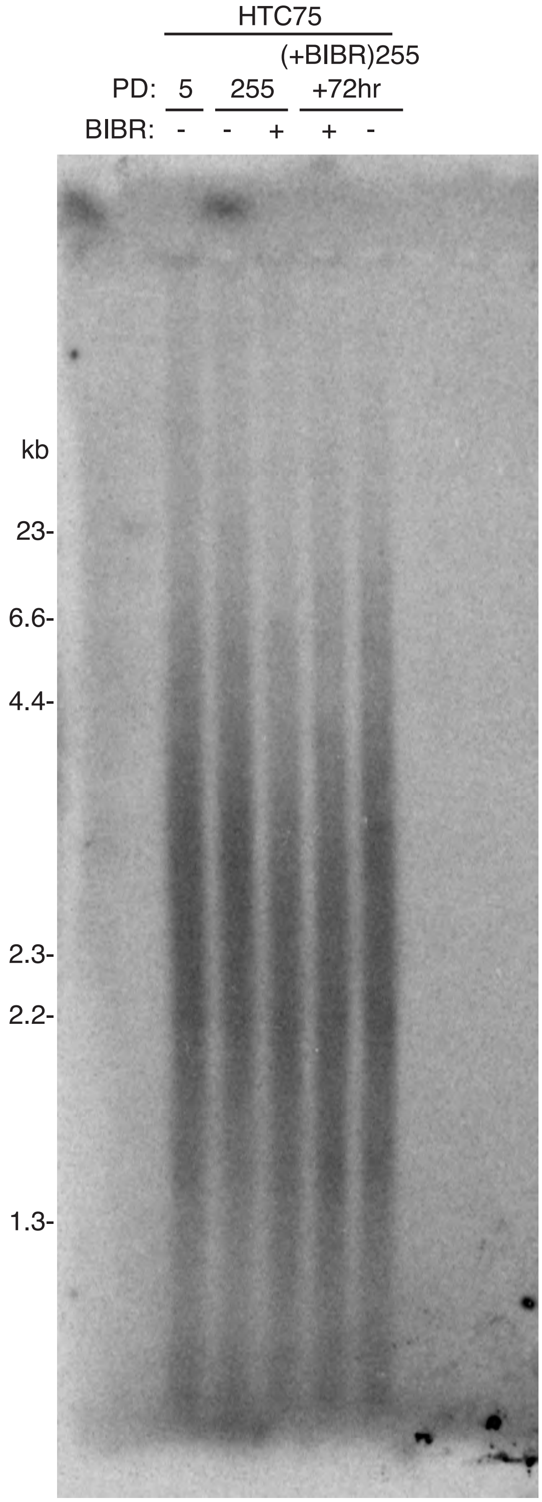

Figure S1F

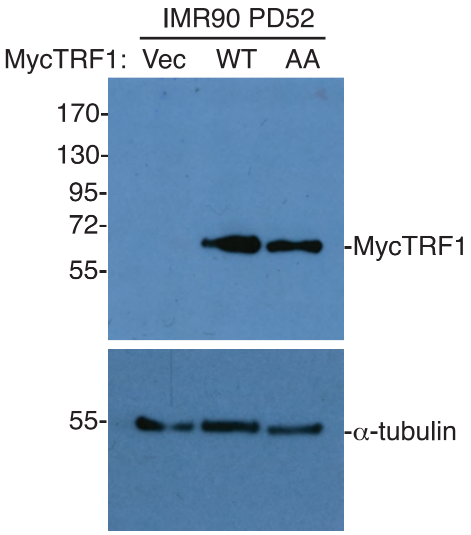
IMR90 PD52
MycTRF1:
Vec
WT
AA
170-
130-
95-
72-
55-
-MycTRF1
55-
-α-tubulin

Figure S2A

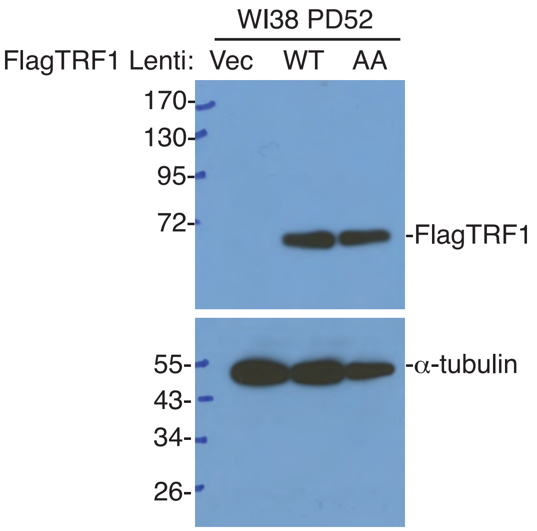

Figure S2B

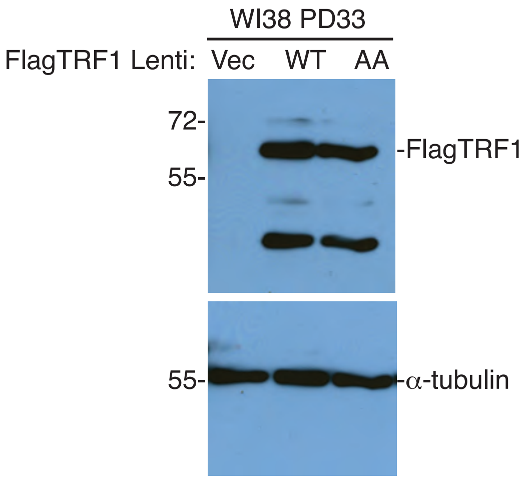

Figure S4F

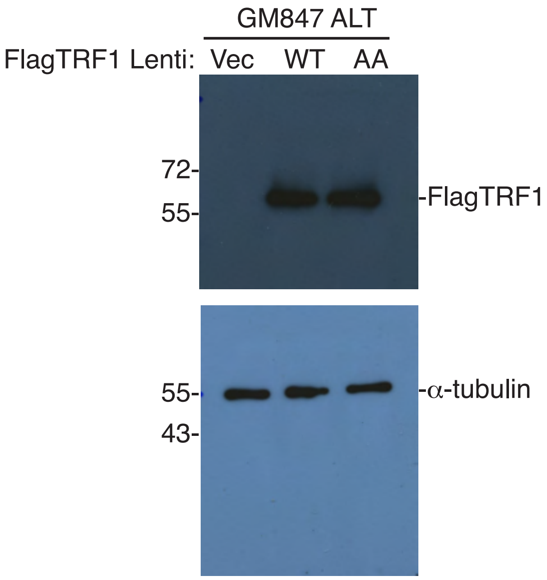
GM847 ALT
FlagTRF1 Lenti:
Vec
WT
AA
72-
55-
-FlagTRF1
55-
43-
-α-tubulin

Figure S5A

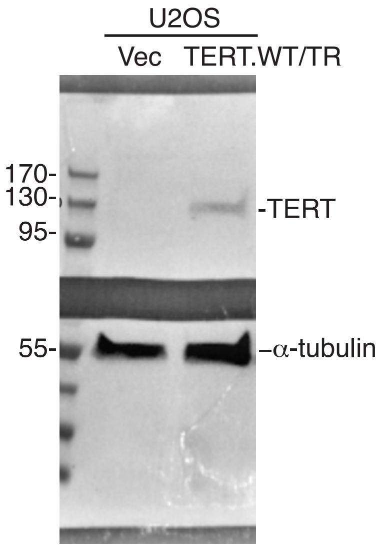

Figure S5E

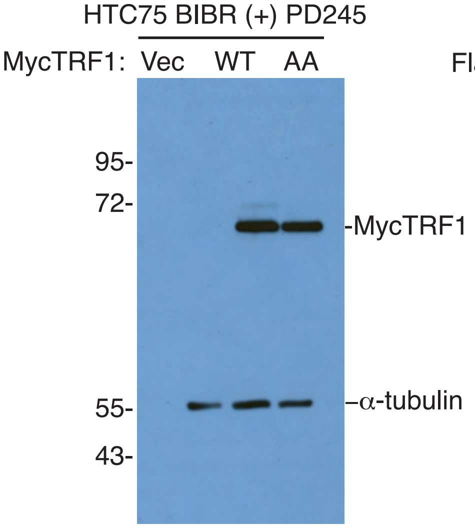

Figure S5F

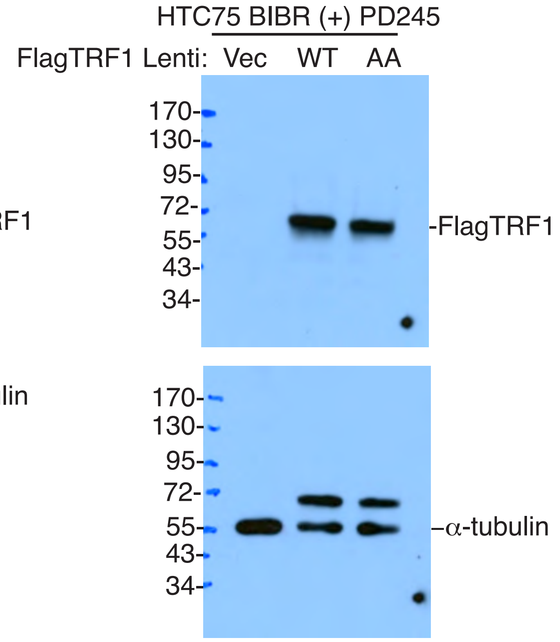

Supplement: Supplementary file 4 — Source Data [file 41467_2020_17133_MOESM4_ESM.zip › SourceDataCroppedBlotsCombinedReduced.pdf]
