# Supplementary material for: Persistent telomere cohesion protects aged cells from premature senescence
Source: Nat Commun. 2020 Jul 3;11:3321. doi: 10.1038/s41467-020-17133-4 (PMC7335080; doi:10.1038/s41467-020-17133-4)
Supplement: Supplementary file 2 — Peer Review [file 41467_2020_17133_MOESM2_ESM.pdf]

Editorial Note: Parts of this peer review file have been redacted as indicated to remove third-party material where no permission to publish could be obtained.

Reviewers' comments:

Reviewer #1 (Remarks to the Author):

This manuscript details a robust study by the Smith lab focusing on the intersection between telomere length and telomere cohesion in aging and ALT cells. The presented experimentation probes how altering telomere cohesion through multiple methods leads to different molecular outcomes at chromosome ends and the resulting functional consequences in pre-senescent/senescent or ALT-positive human cells.

In summary, the authors identify that decreased TRF1 density at shortened telomeres in aged cells results in persistent telomere cohesion. TRF1 over-expression in aged cells promotes resolution of telomere cohesion through tankyrase recruitment (nicely demonstrated through endogenous TRF1 gene editing and expression of mutant TRF1 alleles). Promoting resolution of telomere cohesion in aged cells through TRF1 over-expression conferred an additional sub-telomere recombination phenotype. Critically, the sub-telomere recombination phenotype was suppressed at cohesion resolved telomeres by telomerase-dependent telomere elongation. This key result defines that it is specifically shortened telomeres that are subjected to sub-telomeric recombination when telomere cohesion is resolved. Thereby identifying why similar recombination does not occur at the cohesion resolved telomeres in young or telomerase positive cells. Further, the team identifies sub-telomere recombination in this context is regulated by ATR, CHK1 and RAD51, and independent of RAD52, CHK2 and POLD3. Thus distinguishing the sub-telomere recombination identified here from break-induced telomere synthesis. The surprising result to this reviewer is that the observed sub-telomere recombination drives DNA damage response activation. Recombination typically resolves DNA damage response foci. Regardless, the data demonstrate that suppressing recombination reduces rescues growth arrest in p53-competent U2OS ALT cells and suppresses senescence phenotypes in primary fibroblasts. Finally, in the cell division immediately preceding senescence an increase in telomere cohesion resolution in observed, coinciding with increased sub-telomere recombination. This may contribute to telomere-dependent senescence.

Overall the experiments are well done and the data convincing. The topic is pertinent to those interested in telomere biology, genome maintenance, cohesion, homologous recombination, and cell aging. I have comments that requires addressing in relation to the "telomere copying" and DDR phenotypes. Other comments are cosmetic or clarifying statements that address minor details in the manuscript. Upon revision the manuscript is suitable for publication in Nature Communications.

Major Comments.

1. The readout of sub-telomere recombination phenotype is clear. Further, it is clear this phenotype is dependent on ATR/CHK1/RAD51. However, it is not clear to this reviewer that the authors are observing "telomere copying". Telomere copying would result in duplication of the sub-telomeric region from one chromosome to another. This is suggestive of break-induced telomere synthesis, which is inconsistent with pathway analysis in figure 2A, B. To me, the phenotype looks similar to "telomere fragility". With telomere fragility (still a poorly defined phenotype) the telomere focus becomes spread out and ill-defined, or multiple dots occur at a single chromosome end, but the intensity of the entirety of the telomere signal at that chromosome end is not necessarily diminished. This is like the sub-telomere phenotype shown here (most notably the 13q telomere in Figure 2F and 16p in Fig 4I). What exactly does these probes hybridize to? Is it a repetitive sequence? To show telomere copying occurs it is necessary to perform cytogenetic experiments and demonstrate movement of the sub-telomere tag to another chromosome on a metaphase spread under conditions where sub-telomere recombination is promoted. This experiment will determine if the outcome is inter-chromosome recombination (i.e. copying) or potentially intra-chromosome recombination (sub-telomere fragility?). This may be significant, as it could indicate that an intra-chromosomal telomere

recombination event leads to the unexpected recombination-dependent damage response. Unlike classical HR which resolves DDR foci.

2. Similarly, I do not think it is possible to conclude in Figure 4J that the observed increase is sub-telomere recombination does not the result of supernumerary chromosomes. A common outcome in aged cells after p53 is suppressed is genome duplication and tetraploid cells. Tetrapliody also occurs in SV40 lgT expressing cells. In Supplementary Figure S3 the 2N cells are at an X-axis value of ~ 275 and the 4N cells ~ 550. If genome duplication occured, the 4N peak represents both G2/M phase diploid cells and G1-phase tetraploid cells. The resulting G2/M tetraploid cells would have an 8N DNA peak which in this instance would be an X-axis value twice that of the 4N cells, or ~ 1100. This value is off the chart. However, there is a peak stacked on the edge of the graph that may be that tetraploid G2/M phase cells and the "polyploid" measurement represents S-phase tetraploid cells. It is necessary to redo this flow cytometry with an altered voltage to move the 2N peak to ~ 150, so the resulting DNA peaks for 2N, 4N and 8N are 150, 300 and 600. This will indicate if the observed increase is recombined telomeres are due to genome duplication or more recobination events. If the 8N peak is not observed, a cytogenetic experiment as described in point 1 will definitively demonstrate telomere copying.

3. Is the recombination-dependent DDR observed in figure 3E occurring at the telomeres If so, this would nicely demonstrate that the recombination phenotype at telomeres drives the DDR.

Minor comments.
1. While the data all appear significant, providing stats would be useful for Fig 2A, D, E, G, H; Fig 5H, I; Fig 6G

2. Describing the phenotype as "telomere copying" requires verification as described above. If this proves impossible, "recombination" or some other general term should be applied.

3. Page 8 2nd paragraph. Figure 4B is accidentally called out in place of 5B.

4. It is worth clarifying for the reader that U2OS cells are unique as the only p53-competent ALT cell line. Unless data are presented that demonstrate otherwise, I expect altering telomere cohesion only induces growth arrest in the U2OS ALT line. This will likely not be a universal attribute of ALT which almost always stratifies with a compromised p53 pathway.

5. Page 10, top paragraph. I suggest adding the clarifier to assist reader comprehension putting emphasis that it is the telomere elongation that matter: "Thus, similar to the results described above for ALT cells, telomerase forced resolution of telomere cohesion, but at the same time suppressed subtelomere recombination between the resolved and extended telomeres."

Reviewer #2 (Remarks to the Author):

In this manuscript, Azarm et al. investigated the effect of overexpression of TRF1WT and TRF1 mutants on resolution of persistent telomere cohesion and subtelomere recombination in several cell types. They demonstrated that overexpression of TRF1WT increases resolution of telomere cohesion and subtelomere recombination in aging cells, which is associated with increased DNA damage, RAD51 foci as well as drastic growth arrest. They went on to show that overexpression of TRF1WT also increases resolution of telomere cohesion and subtelomere recombination in ALT cells. In addition, they showed that overexpression of TRF1WT in telomerase-inhibited HTC75 cells rescues resolution of telomere cohesion and increases subtelomere recombination. Furthermore, they showed that loss of persistent telomere cohesion and subtelomere recombination accompany an onset of senescence. The authors propose that telomere erosion that occurs in the absence of telomerase induces persistent

telomere cohesion, which serves as a protective mechanism against subtelomere recombination, DNA damage and premature activation of senescence.

The manuscript presents several major issues as detailed below that raise questions about their model.

1. Although the authors have shown that persistent telomere cohesion is positively correlated with telomere shortening, they have fallen short of demonstrating a direct link that resolution of persistent telomere cohesion causes subtelomere recombination. Work from several groups has implicated TRF1 in regulating homologous recombination (Wang et al 2018 Cell Death Differ; Bower and Griffith 2014 Biochem; McKerlie et al 2013 NAR). A possible scenario is that resolution of persistent telomere cohesion and subtelomere recombination as well as increased DNA damage foci and RAD51 foci in aged cells overexpressing TRF1WT are individual consequences of overexpression of TRF1WT and that persistent telomere cohesion is an independent event from subtelomere recombination.

2. The authors showed that overexpression of TRF1WT causes rapid entry into senescence in U2OS cells, which conflicts with a recent report that overexpression of TRF1WT does not affect cell cycle progression in U2OS cells (Silva et al 2019 Nat Commun). This discrepancy is concerning given that many of key experiments were performed with overexpression of TRF1WT. The authors need to address whether an excessive amount of overexpressed TRF1WT (rather than resolution of persistent telomere cohesion per se) is responsible for causing subtelomere recombination, DNA damage and rapid growth arrest.

3. Presumably the number of TRF1 binding sites in aged WI38 PD52 cells is very limited due to shortened telomeres. One might assume that a vast majority of overexpressed TRF1WT and TRF1AA are not bound to telomeres. Presumably overexpressed unbound TRF1WT would bind tankyrase and soak tankyrase away from telomeres whereas overexpressed unbound TRF1AA would not do so. This scenario would raise questions about their model that TRF1WT aged cells have more tankyrase at telomeres than TRF1AA aged cells. Is tankyrase responsible for the resolution of persistent telomere cohesion in TRF1WT aged cells?

4. TRF1 is essential for ALT activity. Does overexpression of TRF1 affect hallmarks of ALT cells (APBs, C-circles, telomere heterogeneity and T-SCE)? Could misregulation in ALT activity as a result of overexpressed TRF1WT (rather than resolution of persistent telomere cohesion) account for rapid growth arrest seen in ALT U2OS cells?

Other specific issues:

1. In Fig. 1, the authors showed that TRF1G18P, a knockin mutation, leads to persistent telomere cohesion. The authors need to use these TRF1WT and TRF1G18P 293T cell lines to address whether persistent telomere cohesion induced by TRF1G18P expressed at a physiological level serves a protective mechanism against subtelomere recombination, DNA damage and RAD51 foci. Does TRF1G18P affect telomere length and prevent subtelomere recombination, DNA damage foci and RAD51 foci? In addition, does telomerase inhibition cause growth arrest in 293T TRF1WT cells but not in TRF1G18P cells?
Regarding Fig. 1A and 1B, given that the authors suggest that TRF1-mediated tankyrase controls resolution of telomere cohesion, they should demonstrate via ChIPs whether the level of tankyrase at telomeres is affected by TRF1G18P in 293T cells.

2. The authors showed a single cell image in Fig. 1F, claiming that TRF1AA does not recruit tankyrase to telomeres in WI38 PD52 cells. This data from a single cell image is insufficient. A quantitative method such as ChIPs should be used to demonstrate if TRF1AA affects the level of tankyrase at telomeres in aged WI38 PD52 cells.

3. Fig. 3A-3D showed that over 15% of aged WI38 cells overexpressing TRF1WT exhibit >5 RAD51 foci overall whereas less than 8% of them exhibit RAD51 foci at telomeres. Given that a lower threshold was used for scoring cells with RAD51 foci at telomeres (≥2 RAD51/TRF2 colocalizing foci vs >5 RAD51 foci overall), their data suggest that at least half of RAD51 foci as a result of overexpression of TRF1WT in aged WI38 are not associated with telomeres. How specific is the effect of overexpression of TRF1WT on subtelomere recombination in aged WI38 cells? Does overexpression of TRF1WT in aged WI38 cells lead to an increase in overall recombination?
Fig. 3E and 3F showed that overexpression of TRF1WT increased damage foci of gammaH2AX/53BP1 in aged WI38 PD51 cells. Are these damage foci at telomeres or elsewhere in the genome? Is there an increase in chromosome abnormalities, e.g. breaks, fusions etc. in WI38 PD51 cells overexpressing TRF1 WT? Could increased DNA damage be a consequence of increased overall recombination in aged WI38 TRF1WT cells? The authors need to address if overexpression of TRF1 WT causes genome wide DNA damage or telomere-specific DNA damage. In the absence of answers to these questions, it seems premature for them to state "… subtelomere recombination drives the DNA damage".

4. Fig. 4C and 4D showed that overexpression of SV40-LT does not affect persistent telomere cohesion, but increases subtelomere recombination in aged WI38 cells. This data seems to argue against the authors' claim that persistent telomere cohesion affects subtelomere recombination.
In Fig. 4D and 4H, the authors showed that overexpression of SV40-LT increase subtelomere recombination and abrogates growth arrest of TRF1WT WI38 cells. Based on these data, they concluded that loss of p53 permits subtelomere copying and abrogates growth arrest. However, SV40-LT has many targets in addition to p53. The authors need to show if targeting p53 alone increases subtelomere copying and abrogates growth arrest.

5. Fig. 4J showed a further increase in subtelomere coping on Day 4. Is this increase associated with a further reduction in telomere singlets in TRF1 WT WI38 SV40-LT cells on Day 4? In addition, is this increase in subtelomere copying associated with an increase in RAD51 foci overall?

6. The authors need to use a quantitative method such as ChIPs to show that TRF1WT but not TRF1AA increases the level of tankyrase at telomeres in ALT cells. A single cell image shown in Fig. 5B is insufficient.

7. In Fig. 5F, without POLD3 knockdown, about 5% of GM847-TRF1AA cells showed >3 16p foci. In Fig. 5I, knockdown of POLD3 resulted in 30% of GM847-TRF1AA mitotic cells with >3 16p foci (Fig. 5I). Their data suggest that loss of POLD3 increases subtelomere recombination, which contradicts their statement "Subtelomere recombination was independent of POLD3…" on Pg8.

8. In Fig. 5M-5O, overexpression of TRF1WT causes rapid growth arrest in U2OS cells. Does overexpression of TRF1WT cause growth arrest in other ALT cell lines? In addition, does knockdown or knockout of p53 abrogate growth arrest in U2OS TRF1WT cells?

9. The authors claimed that telomerase expression abrogates subtelomere recombination. This statement is not supported by their data shown in Fig. 6D and 6L that telomerase expression or inhibition has no impact on the percentage of mitotic cell with >3 16p loci.

10. In Fig. 6D, introduction of telomerase into GM847 leads to resolution of persistent telomere cohesion but has no effect on subtelomere recombination. This data does not support the idea that persistent telomere cohesion protects against subtelomere recombination.

11. Does overexpression of TRF1WT cause increased DNA damage, RAD51 foci and growth arrest in BIBR-treated HTC75 PD245 cells?

12. The authors suggest that telomere erosion at the last few PDs prior to senescence onset results in loss of telomeres cohesion (singlets). Could this sudden loss of singlets be caused by a sudden

increase in chromosome missegregation? Is there any increase in micronuclei with 16p telo or 13q telo foci at senescence onset?

Reviewer #3 (Remarks to the Author):

This manuscript by Azarm et al. investigates the role of the shelterin component TRF1 in senescence induction. They demonstrate that insufficient binding of TRF1 at shortened telomeres plays a protective role, preventing a recombination induced DNA damage response. Importantly, they show in every cellular context tested – ageing cells, ALT cells, or telomerase positive cells where telomerase is inhibited – that shortened telomeres induce persistent telomere cohesion due to reduced TRF1 binding at telomeres, thereby delaying senescence.

The manuscript is logically laid out and the data are clearly presented. Additionally, the data fully support the conclusions reached except for a couple minor exceptions noted below. The impact and significance of the findings are appropriate for publication for Nature Communications in my opinion.

Only a few minor issues remain to be addressed in my opinion before publication:

1. In the introduction, the sentence “Telomere function is regulated by shelterin, which is recruited to telomeres by the TTAGGG doubled-stranded repeat binding proteins TRF1 and TRF2.” needs to be modified, as TRF1 and TRF2 are actually part of the shelterin complex. As currently stated, it suggests that the 6-member shelterin complex is recruited by TRF1 and TRF2.

2. The authors hypothesize that insufficient TRF1 loading at telomeres in ageing cells results in attenuated recruitment of endogenous tankyrase and a lack of resolution of telomere cohesion. While the authors demonstrate that overexpression of an epitope-tagged version of TRF1 leads to TRF1 loading at telomeres and consequent tankyrase recruitment (Fig. 1F), they never directly demonstrate a decrease in endogenous TRF1 loading at aging cell telomeres. I think this should be addressed experimentally before publication.

3. The data in Fig. 2D, E, G and H appear to be from a single experiment. Replicates are needed for these experiments, and the graphs need to show error bars.

4. Methods under the section Plasmids: “Telomerase was expressing using the pBABE hTERT-hTR plasmid.” Change to “Telomerase was expressed…”.

RESPONSE TO REVIEWERS

We thank the Reviewers for their thoughtful and insightful comments. We have addressed their concerns and feel that the manuscript as a result has been greatly improved.

Reviewers' comments:

Reviewer #1 (Remarks to the Author):

This manuscript details a robust study by the Smith lab focusing on the intersection between telomere length and telomere cohesion in aged and ALT cells. The presented experimentation probes how altering telomere cohesion through multiple methods leads to different molecular outcomes at chromosome ends and the resulting functional consequences in pre-senescent/senescent or ALT-positive human cells.

In summary, the authors identify that decreased TRF1 density at shortened telomeres in aged cells results in persistent telomere cohesion. TRF1 over-expression in aged cells promotes resolution of telomere cohesion through tankyrase recruitment (nicely demonstrated through endogenous TRF1 gene editing and expression of mutant TRF1 alleles). Promoting resolution of telomere cohesion in aged cells through TRF1 over-expression conferred an additional sub-telomere recombination phenotype. Critically, the sub-telomere recombination phenotype was suppressed at cohesion resolved telomeres by telomerase-dependent telomere elongation. This key result defines that it is specifically shortened telomeres that are subjected to sub-telomeric recombination when telomere cohesion is resolved. Thereby identifying why similar recombination does not occur at the cohesion resolved telomeres in young or telomerase positive cells. Further, the team identifies sub-telomere recombination in this context
is regulated by ATR, CHK1 and RAD51, and independent of RAD52, CHK2 and POLD3. Thus, distinguishing the sub-telomere recombination identified here from break-induced telomere synthesis. The surprising result to this reviewer is that the observed sub-telomere recombination drives DNA damage response activation. Recombination typically resolves DNA damage response foci. Regardless, the data demonstrate that suppressing recombination reduces rescues growth arrest in p53-competent U2OS ALT cells and suppresses senescence phenotypes in primary fibroblasts. Finally, in the cell division immediately preceding senescence an increase in telomere cohesion resolution in observed, coinciding with increased sub-telomere recombination. This may contribute to telomere-dependent senescence.

Overall the experiments are well done and the data convincing. The topic is pertinent to those interested in telomere biology, genome maintenance, cohesion, homologous recombination, and cell aged. I have comments that requires addressing in relation to the "telomere copying" and DDR phenotypes. Other comments are cosmetic or clarifying statements that address minor details in the manuscript. Upon revision the manuscript is suitable for publication in Nature Communications.

Major Comments.
1. The readout of sub-telomere recombination phenotype is clear. Further, it is clear this phenotype is dependent on ATR/CHK1/RAD51. However, it is not clear to this reviewer that the authors are observing "telomere copying". Telomere copying would result in duplication of the sub-telomeric region from one chromosome to another. This is suggestive of break-induced telomere synthesis, which is inconsistent with pathway analysis in figure 2A, B. To me, the phenotype looks similar to "telomere fragility". With telomere fragility (still a poorly defined phenotype) the telomere focus becomes spread out and ill-defined, or multiple dots occur at a single chromosome end, but the intensity of the entirety of the telomere signal at that chromosome end is not necessarily diminished. This is like the sub-telomere phenotype shown here (most notably the 13q telomere in Figure 2F and 16p in Fig 4I). What exactly does these probes hybridize to? Is it a repetitive sequence?

The probes (purchased from Cytocell) are generated against the chromosome specific unique sequences adjacent to the telomere and telomere-associated repeats. The unique region is ~100 kb and it varies in its distance from the end, depending on the chromosome.

Regarding telomere fragility, this phenotype has been observed using TTAGGG-repeat FISH probes, but not using subtelomere probes (as far as we know). However, as the Reviewer points out, we do observe small dots next to (and often connected to) larger signals. **We do not score the small dots as copying**. In the example the reviewer cites in 4I, the "fragile" looking signals (the small dots next to doublets) are not scored as copying. They are considered as part of a single 16p locus.

Prompted by the reviewer's concerns, we examined our data (for example in Fig. 1I and J where we established the copying phenotype) to determine the frequency of the small dots and whether they were influenced by overexpression of TRF1. We observed them at a frequency of approximately 16% and the frequency was similar in all samples (Vec, TRF1.WT, and TRF1.AA). Thus, we observe small dots, but we do not score them and their frequency is not influenced by our experimental conditions. Finally, regarding the other example the reviewer cites (Fig. 2F) we agree that one signal in the doublet is smaller than the other but, it is not connected and not so small as to be a "fragile". Nonetheless, we have replaced the figure with more representative images where the doublet signals are similar in size (new Fig. 2F).

To show telomere copying occurs it is necessary to perform cytogenetic experiments and demonstrate movement of the sub-telomere tag to another chromosome on a metaphase spread under conditions where sub-telomere recombination is promoted. This experiment will determine if the outcome is inter-chromosome recombination (i.e. copying) or potentially intra-chromosome recombination (sub-telomere fragility?). This may be significant, as it could indicate that an intra-chromosomal telomere recombination event leads to the unexpected recombination-dependent damage response. Unlike classical HR which resolves DDR foci.

We now provide new data using cytogenetic experiments to demonstrate that the subtelomere sequence has moved to another chromosome on a metaphase spread. In new Figures S4B – S4E, we show metaphase spread analysis and provide quantification showing a significant increase in metaphase spreads exhibiting subtelomere copying of the (S4B-C)16p or (S4D-E)13q subtelomere to another chromosome in U2OS cells transfected with TRF1.WT, compared to TRF1.AA or a vector control.

2. Similarly, I do not think it is possible to conclude in Figure 4J that the observed increase is sub-telomere recombination does not the result of supernumerary chromosomes. A common outcome in aged cells after p53 is suppressed is genome duplication and tetraploid cells. Tetrapliody also occurs in SV40 lgT expressing cells. In Supplementary Figure S3 the 2N cells are at an X-axis value of ~ 275 and the 4N cells ~ 550. If genome duplication occured, the 4N peak represents both G2/M phase diploid cells and G1-phase tetraploid cells. The resulting G2/M tetraploid cells would have an 8N DNA peak which in this instance would be an X-axis value twice that of the 4N cells, or ~ 1100. This value is off the chart. However, there is a peak stacked on the edge of the graph that may be that tetraploid G2/M phase cells and the "polyploid" measurement represents S-phase tetraploid cells. It is necessary to redo this flow cytometry with an altered voltage to move the 2N peak to ~ 150, so the resulting DNA peaks for 2N, 4N and 8N are 150, 300 and 600. This will indicate if the observed increase is recombined telomeres are due to genome duplication or more recobination events. If the 8N peak is not observed, a cytogenetic experiment as described in point 1 will definitively demonstrate telomere copying.

We have redone the flow cytometry with an altered voltage to move the 2N peak to ~150, so the resulting DNA peaks for 2N, 4N and 8N are 150, 300 and 600, respectively. As shown in new Fig. S3, we do not detect an 8N peak. Thus, the increase in subtelomere recombination that we observe is not due to tetraploidy. Therefore, as the reviewer states, the lack of an 8N peak, combined with new cytogenetic experiments (new Fig. S4B-D) described above definitively demonstrates telomere copying.

3. Is the recombination-dependent DDR observed in figure 3E occurring at the telomeres If so, this would nicely demonstrate that the recombination phenotype at telomeres drives the DDR.

Yes, the DNA damage occurs at the telomeres. We performed double immunofluorescence analysis on aged WI38 cells overexpressing Vector, TRF1.WT, or TRF1.AA using γH2AX to mark DNA damage foci and TIN2 to mark telomeres. We show a significant (3-fold) increase in γH2AX/TIN2 colocalizing foci in TRF1.WT compared to Vector or TRF1.AA (new Fig. 3G and 3H).

Minor comments.
1. While the data all appear significant, providing stats would be useful for Fig 2A, D, E, G, H; Fig 5H, I; Fig 6G

We have now provided stats for Fig 2A, D, E, G, H; Fig 5H, I; Fig 6G.

2. Describing the phenotype as "telomere copying" requires verification as described above. If this proves impossible, "recombination" or some other general term should be applied.

We have verified the phenotype at telomere copying as described above.

3. Page 8 2nd paragraph. Figure 4B is accidentally called out in place of 5B.

Corrected.

4. It is worth clarifying for the reader that U2OS cells are unique as the only p53-competent ALT cell line. Unless data are presented that demonstrate otherwise, I expect altering telomere cohesion only induces growth arrest in the U2OS ALT line. This will likely not be a universal attribute of ALT which almost always stratifies with a compromised p53 pathway.

We have now clarified that U2OS is "a checkpoint proficient ALT cell line with wild-type p53". Furthermore, we have now performed growth curve analysis in GM847, an ALT cell line lacking p53 checkpoint function and provide new data (Fig. S4F and S4G) to show that in these checkpoint-deficient ALT cells TRF1.WT does not induce a growth arrest. Thus, as the reviewer suggests, the growth arrest is not a general attribute of ALT.

5. Page 10, top paragraph. I suggest adding the clarifier to assist reader comprehension putting emphasis that it is the telomere elongation that matter: "Thus, similar to the results described above for ALT cells,

telomerase forced resolution of telomere cohesion, but at the same time suppressed subtelomere recombination between the resolved and extended telomeres."

We have added the clarifier.

Reviewer #2 (Remarks to the Author):

In this manuscript, Azarm et al. investigated the effect of overexpression of TRF1WT and TRF1 mutants on resolution of persistent telomere cohesion and subtelomere recombination in several cell types. They demonstrated that overexpression of TRF1WT increases resolution of telomere cohesion and subtelomere recombination in aged cells, which is associated with increased DNA damage, RAD51 foci as well as drastic growth arrest. They went on to show that overexpression of TRF1WT also increases resolution of telomere cohesion and subtelomere recombination in ALT cells. In addition, they showed that overexpression of TRF1WT in telomerase-inhibited HTC75 cells rescues resolution of telomere cohesion and increases subtelomere recombination. Furthermore, they showed that loss of persistent telomere cohesion and subtelomere recombination accompany an onset of senescence. The authors propose that telomere erosion that occurs in the absence of telomerase induces persistent telomere cohesion, which serves as a protective mechanism against subtelomere recombination, DNA damage and premature activation of senescence.

The manuscript presents several major issues as detailed below that raise questions about their model.

1. Although the authors have shown that persistent telomere cohesion is positively correlated with telomere shortening, they have fallen short of demonstrating a direct link that resolution of persistent telomere cohesion causes subtelomere recombination. Work from several groups has implicated TRF1 in regulating homologous recombination (Wang et al 2018 Cell Death Differ; Bower and Griffith 2014 Biochem; McKerlie et al 2013 NAR). A possible scenario is that resolution of persistent telomere cohesion and subtelomere recombination as well as increased DNA damage foci and RAD51 foci in aged cells overexpressing TRF1WT are individual consequences of overexpression of TRF1WT and that persistent telomere cohesion is an independent event from subtelomere recombination.

We have shown through a number of assays in this paper (and previously) that persistent telomere cohesion is mediated by the RGCADG tankyrase binding site at amino acid (aa) position 13 in the amino terminal acidic domain of TRF1. This domain is distinct from all other functional domains of TRF1: the TRF1 homology domain (aa 67-264), the linker domain (aa 264-379) and the DNA-binding myb domain (aa 379-439). All of our experiments are performed with TRF1.WT versus TRF1.AA. TRF1.AA is expressed at the same level as WT and it has all the other functional domains of TRF1 intact. The demonstration that TRF1.AA does not induce any of the phenotypes (and generally behaves like the vector control) indicates that the tankyrase binding site and not the other domains of TRF1 is responsible.

Additionally, below we specifically address the papers cited by the Reviewer:

Wang et al 2018 Cell Death Differ. "Dual roles of TRF1 in tethering telomeres to the nuclear envelope and protecting them from fusion during meiosis". This work addresses the action of TRF1 in the nuclear envelope in meiosis. TRF1 function here depends on its interaction with a meiosis specific protein TERB1 that heterodimerizes with TRF1 through the TRF1 homology domain (aa 67-264). This is a meiotic

phenotype. It is mediated by the homology domain which is intact in TRF1.WT and AA and thus cannot account for the differential effects of TRF1.WT versus TRF1.AA.

McKerlie et al 2013 NAR. "Phosphorylated (pT371)TRF1 is recruited to sites of DNA damage to facilitate homologous recombination and checkpoint activation". This work addresses the function of a phosphorylation site at amino acid 371 in the linker domain. This site is intact in TRF1.WT and AA and thus cannot account for the differential effects of TRF1.WT versus TRF1.AA.

Bower and Griffith 2014 Biochem. "TRF1 and TRF2 Differentially Modulate Rad51-Mediated Telomeric and Nontelomeric Displacement Loop Formation in Vitro". This work performs analyses in vitro with purified proteins. They show that Rad51 can form a complex with telomeric DNA (measured as 15% of input). They show that addition of purified TRF1 stimulates it (measured as 18% of input). This may be interesting however, we feel that since it is an in vitro analysis and the effect is relatively minor, it has little bearing on the robust effects that we observe in human cells with TRF1.WT versus AA.

2. The authors showed that overexpression of TRF1WT causes rapid entry into senescence in U2OS cells, which conflicts with a recent report that overexpression of TRF1WT does not affect cell cycle progression in U2OS cells (Silva et al 2019 Nat Commun). This discrepancy is concerning given that many of key experiments were performed with overexpression of TRF1WT. The authors need to address whether an excessive amount of overexpressed TRF1WT (rather than resolution of persistent telomere cohesion per se) is responsible for causing subtelomere recombination, DNA damage and rapid growth arrest.

In the Silva paper, the authors infect cells with TRF1 retrovirus and then 5 days later transfect with control siRNA. They then harvest cells 2 days later (total of 1 week), perform FACS analysis, and show that the FACS profiles between TRF1 infected and control infected U2OS cells are similar. This is different from our experiments in Fig. 5N and O. First, we did not perform FACS analysis. We infected cells with lentiviruses and then analyzed for β-gal associated senescence 2 days later. We showed an increase in β-gal positive cells: 30% in TRF1.WT, compared to 6-8% in vector or TRF1.AA cells. We performed growth curve analysis and showed that the cell numbers in TRF1.WT do not increase through Day 5. The effect is specific to TRF1.WT (it does not occur with TRF1.AA overexpression) and thus is not a general effect of TRF1 overexpression. We cannot say what the FACS analysis would look like at Day 7. It is possible that the remaining non-senescing ALT cancer cells would outgrow the senescing cells and give a similar profile as control cells. Altogether, we have done very different experiments from the Silva paper and do not see our results as in conflict.

3. Presumably the number of TRF1 binding sites in aged WI38 PD52 cells is very limited due to shortened telomeres. One might assume that a vast majority of overexpressed TRF1WT and TRF1AA are not bound to telomeres. Presumably overexpressed unbound TRF1WT would bind tankyrase and soak tankyrase away from telomeres whereas overexpressed unbound TRF1AA would not do so. This scenario would raise questions about their model that TRF1WT aged cells have more tankyrase at telomeres than TRF1AA aged cells. Is tankyrase responsible for the resolution of persistent telomere cohesion in TRF1WT aged cells?

Yes, we published previously (Kim and Smith, MBC 2014) that overexpression of tankyrase 1 forces resolution of persistent telomere cohesion in aged cells, indicating that tankyrase is responsible for resolution of persistent telomere cohesion in aged cells with wild type TRF1.

4. TRF1 is essential for ALT activity. Does overexpression of TRF1 affect hallmarks of ALT cells (APBs, C-circles, telomere heterogeneity and T-SCE)? Could misregulation in ALT activity as a result of

overexpressed TRF1WT (rather than resolution of persistent telomere cohesion) account for rapid growth arrest seen in ALT U2OS cells?

We measured the frequency of APBs (a hallmark of the ALT phenotype) in U2OS cells transfected with Vector, TRF1.WT, or TRF1.AA and scored one hundred cells each for cells with greater than 2 APBs and observed no significant difference: 16%, 15%, and 16% APBs for Vector, TRF1.WT, and TRF1.AA cells, respectively.

Our data are consistent with overexpression of TRF1 and the resulting resolution of cohesion (rather than misregulation of ALT activity) accounting for the rapid induction of senescence. We observe the same result (induction of premature senescence by TRF1.WT but not TRF.AA in (non-ALT) aged cells. The effect is only observed for TRF1.WT not AA. We have shown previously that overexpression of tankyrase 1 forces resolution of persistent telomere cohesion in ALT cells and inhibits cell growth in U2OS cells (Ramamoorthy and Smith, Cancer Cell 2015), indicating that tankyrase 1 is responsible for resolution of persistent telomere cohesion in ALT cells. The demonstration that the senescence growth arrest is induced by TRF1.WT but not TRF1.AA supports the model that the phenotype results from forced resolution of cohesion by tankyrase 1 binding to the tankyrase binding site in the overexpressed wild type TRF1 at telomeres.

Other specific issues:

1. In Fig. 1, the authors showed that TRF1G18P, a knockin mutation, leads to persistent telomere cohesion. The authors need to use these TRF1WT and TRF1G18P 293T cell lines to address whether persistent telomere cohesion induced by TRF1G18P expressed at a physiological level serves a protective mechanism against subtelomere recombination, DNA damage and RAD51 foci. Does TRF1G18P affect telomere length and prevent subtelomere recombination, DNA damage foci and RAD51 foci?

The purpose of the TRF1G18P knockin was to demonstrate that the tankyrase binding site in TRF1 is essential for resolution of telomere cohesion and thereby validate our approach of using TRF1.WT versus a TRF1 tankyrase-binding site mutant as a control in all of our experiments. 293T cells are telomerase positive and therefore do not need protection against subtelomere recombination, DNA damage, and RAD51 foci. We show that subtelomere recombination (and its consequences) only occur in cells that have critically short telomeres and lack telomerase.
In addition, does telomerase inhibition cause growth arrest in 293T TRF1WT cells but not in TRF1G18P cells?

We did not observe a growth arrest in telomerase inhibitor-treated HTC75 TRF1.WT cells.

Regarding Fig. 1A and 1B, given that the authors suggest that TRF1-mediated tankyrase controls resolution of telomere cohesion, they should demonstrate via ChIPs whether the level of tankyrase at telomeres is affected by TRF1G18P in 293T cells.

We have shown previously using quantitative ChIP analysis (Bisht et al, MCB 2012; pasted below for the Reviewer) that TRF1.WT but not TRF1.AA recruits endogenous tankyrase to telomeres. These experiments were performed in HeLaI.2.11 cells, a clonal cell line that has extremely long telomeres that are homogenous in length. This analysis is technically challenging as tankyrase 1 is low abundant at telomeres and does not bind directly to telomeric DNA. Detection by ChIP requires long homogeneous telomeres and overexpression of TRF1. We are not able to detect endogenous tankyrase at telomeres by ChIP in cells with short and/or heterogenous telomeres, such as 293T, the cell line with the CRISPRed in TRF1

point mutation. The purpose of generating the TRF1G18P mutant was to validate the dependence of the persistent cohesion phenotype on tankyrase recruitment to telomeres by TRF1. We think that the observation that a CRISPR-generated point mutation in the tankyrase binding site of endogenous TRF1 leads to persistent telomere cohesion in three independent clonal cell lines (Fig. 1A and B) clearly demonstrates
that the tankyrase binding site in TRF1 is essential for resolution of cohesion.

2. The authors showed a single cell image in Fig. 1F, claiming that TRF1AA does not recruit tankyrase to telomeres in WI38 PD52 cells. This data from a single cell image is insufficient. A quantitative method such as ChIPs should be used to demonstrate if TRF1AA affects the level of tankyrase at telomeres in aged WI38 PD52 cells.

As described above, it is difficult to detect tankyrase with ChIP, particularly in aged cells with short telomeres. That is why we use immunofluorescence – so that we can visualize recruitment directly on a cell to cell basis. To address the reviewer's concern "that data from a single cell image is insufficient", we provide new data where we score approximately 100 cells each in two independent experiments and show in a new Figure S1D that 86% of MycTRF1.WT cells (compared to 1% Myc TRF1.AA cells) recruit endogenous tankyrase 1 to nuclear foci.

3. Fig. 3A-3D showed that over 15% of aged WI38 cells overexpressing TRF1WT exhibit >5 RAD51 foci overall whereas less than 8% of them exhibit RAD51 foci at telomeres. Given that a lower threshold was used for scoring cells with RAD51 foci at telomeres (≥2 RAD51/TRF2 colocalizing foci vs >5 RAD51 foci overall), their data suggest that at least half of RAD51 foci as a result of overexpression of TRF1WT in aged WI38 are not associated with telomeres.

We would not conclude that "at least half of RAD51 foci as a result of overexpression of TRF1.WT in aged WI38 are not associated with telomeres". The critically short telomeres are difficult to detect because they have limited TTAGGG repeats. Thus, just because a Rad51 focus does not colocalize with a TRF1-signal does not necessarily mean that it is not at a telomere. We also point out that the fold increase in % cells with >2 TRF2 colocalizing foci is greater than the fold increase in % cells with >5 Rad51 foci, 5.0 versus 3.3 respectively. It is significant and specific to TRF1.WT.

How specific is the effect of overexpression of TRF1WT on subtelomere recombination in aged WI38 cells? Does overexpression of TRF1WT in aged WI38 cells lead to an increase in overall recombination?

We showed that the recombination was specific to telomeres in aged cells at their final passage; recombination occurred between the 13q subtelomere, but not the 13q arm (FISH; Fig. 7C-E and 7H-I). We now provide new data in Figure S1I and S1L where we introduce Vector, TRF1.WT, and TRF1.AA into aged WI38 cells and perform FISH with the dual 13q subtelomere/arm probe. We show that overexpression of TRF1.WT (but not vector or TRF1.AA) leads to an increase in sub telomere recombination (13q subtelomere probe) and not in overall recombination (13q arm probe).

Fig. 3E and 3F showed that overexpression of TRF1WT increased damage foci of gammaH2AX/53BP1 in aged WI38 PD51 cells. Are these damage foci at telomeres or elsewhere in the genome? Is there an increase in chromosome abnormalities, e.g. breaks, fusions etc. in WI38 PD51 cells overexpressing TRF1 WT? Could increased DNA damage be a consequence of increased overall recombination in aged WI38 TRF1WT cells? The authors need to address if overexpression of TRF1 WT causes genome wide DNA damage or telomere-specific DNA damage. In the absence of answers to these questions, it seems premature for them to state "… subtelomere recombination drives the DNA damage".

We provide new data to show that the overexpression of TRF1.WT causes telomere specific DNA damage. We performed double immunofluorescence analysis on aged WI38 cells overexpressing Vector, TRF1.WT, or TRF1.AA using γH2AX to mark DNA damage foci and TIN2 to mark telomeres. We show a significant (3-fold) increase in γH2AX/TIN2 colocalizing foci in TRF1.WT compared to Vector or TRF1.AA (new Fig. 3G and 3H).

4. Fig. 4C and 4D showed that overexpression of SV40-LT does not affect persistent telomere cohesion, but increases subtelomere recombination in aged WI38 cells. This data seems to argue against the authors' claim that persistent telomere cohesion affects subtelomere recombination.

The observed increase subtelomere recombination was unexpected. The observation suggested to us (as stated on page 8) "that loss of the checkpoint permits a low level of subtelomere copying even with persistent cohesion." This is what prompted us to monitor recombination 4 days after SV40-LT infection to determine if we would see much more recombination in the absence of p53 and we did (Fig. 4H-J).

In Fig. 4D and 4H, the authors showed that overexpression of SV40-LT increase subtelomere recombination and abrogates growth arrest of TRF1WT WI38 cells. Based on these data, they concluded that loss of p53 permits subtelomere copying and abrogates growth arrest. However, SV40-LT has many targets in addition to p53. The authors need to show if targeting p53 alone increases subtelomere copying and abrogates growth arrest.

Previous studies have shown that telomere directed senescence in human cells can be mediated independently by the p53 and p16/RB pathways. Our goal was not to dissect the pathway, but rather to show that the arrest was checkpoint mediated. Indeed, that is why we used SV40-LT to ensure removal of both pathways. We agree with the reviewer we cannot claim a "p53-dependent arrest. We have changed the language throughout the paper and now refer to it as a "checkpoint-dependent arrest"

5. Fig. 4J showed a further increase in subtelomere coping on Day 4. Is this increase associated with a further reduction in telomere singlets in TRF1 WT WI38 SV40-LT cells on Day 4? In addition, is this increase in subtelomere copying associated with an increase in RAD51 foci overall?

We tried to determine if there was a reduction in telomere singlets in TRF1 WT WI38 SV40-LT cells on Day 4. However, we were not able to get a clean readout due to the dramatic increase in singlets resulting from subtelomere copying. We do not see a big increase in RAD51 foci overall from Day1 to Day4, but we would not have anticipated such an increase. We are not proposing that there is more RAD51 and more recombination at Day 4. Rather, we suggest that by removing the checkpoint, the cells do not senesce and therefore they can continue to grow and recombine.

6. The authors need to use a quantitative method such as ChIPs to show that TRF1WT but not TRF1AA increases the level of tankyrase at telomeres in ALT cells. A single cell image shown in Fig. 5B is insufficient.

To address the reviewer's concern that data from "a single cell image is insufficient", we provide new data where we score approximately 100 cells each in two independent experiments and show in a new Figure S4A that 89% of MycTRF1.WT cells (compared to 1% Myc TRF1.AA cells) recruit endogenous tankyrase 1 to nuclear foci.

7. In Fig. 5F, without POLD3 knockdown, about 5% of GM847-TRF1AA cells showed >3 16p foci. In Fig. 5I, knockdown of POLD3 resulted in 30% of GM847-TRF1AA mitotic cells with >3 16p foci (Fig. 5I). Their data suggest that loss of POLD3 increases subtelomere recombination, which contradicts their statement "Subtelomere recombination was independent of POLD3…" on Pg8.

This was a typo. Fig. 5I (like Fig. 5H) was analyzing the effect of (-/+) siPOLD3 in TRF1.WT cells (not AA cells). It has been corrected. We are grateful to the reviewer for catching this error.

8. In Fig. 5M-5O, overexpression of TRF1WT causes rapid growth arrest in U2OS cells. Does overexpression of TRF1WT cause growth arrest in other ALT cell lines? In addition, does knockdown or knockout of p53 abrogate growth arrest in U2OS TRF1WT cells?

We chose U2OS because they are a checkpoint proficient ALT cell line with wild-type p53. We have now performed growth curve analysis in GM847, an ALT cell line lacking p53 checkpoint function and provide new data (Fig. S4F and S4G) to show that in these checkpoint-deficient ALT cells TRF1.WT does not induce a growth arrest.

9. The authors claimed that telomerase expression abrogates subtelomere recombination. This statement is not supported by their data shown in Fig. 6D and 6L that telomerase expression or inhibition has no impact on the percentage of mitotic cell with >3 16p loci.

We claim that telomerase expression abrogates subtelomere recombination **induced by TRF1.WT overexpression.**

In Fig. 6D, we are not inducing subtelomere recombination by TRF1.WT overexpression, we are just introducing telomerase into ALT cells. We show that telomerase forces resolution of cohesion (6C) but does not lead to an increase in subtelomere recombination (6D). This experiment demonstrates (using telomerase negative ALT cells) that short telomeres are responsible for the persistent cohesion phenotype and further that resolution of cohesion may be necessary but it is not sufficient to induce subtelomere copying – for that you need critically shortened telomeres in the absence of telomerase.

In Fig 6L, we are not inducing subtelomere recombination by TRF1.WT overexpression, we are just introducing telomerase (by removing the telomerase inhibitor) into HTC75 cells which exhibit persistent telomere cohesion as a result of being treated for 245 PD with telomerase inhibitor to induce short telomeres. We show that telomerase forces resolution of cohesion (6K) but does not lead to an increase in subtelomere recombination (6L). This experiment demonstrates (using telomerase positive HTC75 cells) that the short telomeres are responsible for the persistent cohesion phenotype and further that resolution of cohesion may be necessary but it is not sufficient to induce subtelomere copying – for that you need critically shortened telomeres in the absence of telomerase

10. In Fig. 6D, introduction of telomerase into GM847 leads to resolution of persistent telomere cohesion but has no effect on subtelomere recombination. This data does not support the idea that persistent telomere cohesion protects against subtelomere recombination.

We claim that persistent telomere cohesion protects against subtelomere recombination **under conditions where telomeres are critically short and in the absence of telomerase.** In Fig. 6D telomerase is expressed, thus we would not anticipate subtelomere recombination. See extended explanation above for Point 9.

11. Does overexpression of TRF1WT cause increased DNA damage, RAD51 foci and growth arrest in BIBR-treated HTC75 PD245 cells?

The purpose of the BIBR-treated HTC75 PD245 cell experiment was to create an artificial setup to demonstrate that short telomeres per se combined with the absence of telomerase promotes persistent telomere cohesion and supports subtelomere copying upon TRF1.WT overexpression. We feel that to take this further with DNA damage, RAD51, and growth curve analysis does not further contribute to our understanding of the mechanism.

12. The authors suggest that telomere erosion at the last few PDs prior to senescence onset results in loss of telomeres cohesion (singlets). Could this sudden loss of singlets be caused by a sudden increase in chromosome missegregation? Is there any increase in micronuclei with 16p telo or 13q telo foci at senescence onset?

We score cells in mitosis and we score the status of two 13q telo loci. If we lost a singlet due to missegregation, then the cell would have only one remaining 13q locus. We do not see aged mitotic cells with only one 13q locus.

Reviewer #3 (Remarks to the Author):

This manuscript by Azarm et al. investigates the role of the shelterin component TRF1 in senescence induction. They demonstrate that insufficient binding of TRF1 at shortened telomeres plays a protective role, preventing a recombination induced DNA damage response. Importantly, they show in every cellular context tested – ageing cells, ALT cells, or telomerase positive cells where telomerase is inhibited – that shortened telomeres induce persistent telomere cohesion due to reduced TRF1 binding at telomeres, thereby delaying senescence.

The manuscript is logically laid out and the data are clearly presented. Additionally, the data fully support the conclusions reached except for a couple minor exceptions noted below. The impact and significance of the findings are appropriate for publication for Nature Communications in my opinion.

Only a few minor issues remain to be addressed in my opinion before publication:

1. In the introduction, the sentence "Telomere function is regulated by shelterin, which is recruited to telomeres by the TTAGGG doubled-stranded repeat binding proteins TRF1 and TRF2." needs to be modified, as TRF1 and TRF2 are actually part of the shelterin complex. As currently stated, it suggests that the 6-member shelterin complex is recruited by TRF1 and TRF2.

We now say: "Telomere function is regulated by the TTAGGG doubled-stranded repeat binding shelterin subunits TRF1 and TRF2."

2. The authors hypothesize that insufficient TRF1 loading at telomeres in ageing cells results in attenuated recruitment of endogenous tankyrase and a lack of resolution of telomere cohesion. While the authors demonstrate that overexpression of an epitope-tagged version of TRF1 leads to TRF1 loading at telomeres and consequent tankyrase recruitment (Fig. 1F), they never directly demonstrate a decrease in endogenous TRF1 loading at aged cell telomeres. I think this should be addressed experimentally before publication.

Previous studies using HeLa cell lines with long versus short telomeres used indirect immunofluorescence analysis to demonstrate that less TRF1 signal was detected at short telomeres (Smogorzewska et al, (2000) MCB 20(5):1659-1668). We now have used the same approach with aged cells. We provide new data using immunofluorescence analysis (new Fig. S1C) using anti-TRF1 versus anti-centromere (ACA) antibodies (as a control that does not change as cells age) to examine young versus old WI38 cells in parallel. Immunofluorescent images were captured and reproduced under the same conditions and settings. As shown in Fig. S1C, we detect a decrease in endogenous TRF1 (but not ACA) at aged cell telomeres.

3. The data in Fig. 2D, E, G and H appear to be from a single experiment. Replicates are needed for these experiments, and the graphs need to show error bars.

We provide new data where we performed replicates and the graphs in Fig. 2D, E, G, and H now have error bars.

4. Methods under the section Plasmids: "Telomerase was expressing using the pBABE hTERT-hTR plasmid." Change to "Telomerase was expressed…".

Corrected.

Reviewers' comments:

Reviewer #1 (Remarks to the Author):

Thank you to the authors for effectively addressing all my queries. I support publication of the revised manuscript.

Reviewer #2 (Remarks to the Author):

The authors have made efforts to address some of the concerns raised previously, however several main issues remain outstanding. This dampens one's enthusiasm to endorse it for publication as it currently stands.

1. The TRF1AA work cited by the authors demonstrates that the tankyrase binding site of TRF1 is responsible for the observed phenotypes (resolution of persistent telomere cohesion, subtelomere recombination) in aged cells overexpressing TRF1WT, but it does not demonstrate the causal relationship between the phenotypes. As a result, the authors have not convincingly shown that there is a causal relationship among phenotypes caused by overexpression of TRF1WT in aged cells. In the absence of experimental evidence for a demonstrated causal relationship between persistent telomere cohesion and subtelomere recombination, their conclusion that persistent telomere cohesion protects against inappropriate recombination is not sufficiently supported.

2. The authors showed that overexpression of TRF1WT in U2OS cells induces DNA damage and cellular senescence, which is in stark contrast to the published report by Silva et al that showed that overexpression of TRF1WT in U2OS does not affect cell cycle profile. The authors attributed this discrepancy to the difference in their ways of doing experiments, e.g. through Day 5 vs Day 7 by Silva et al. It is rather inadequate that the authors just stated that "we cannot say what the FACS analysis would look like at Day 7". Given that their data in Fig. 5O showed that 30% of TRF1WT-overexpressed cells are senesced, it is difficult to imagine that the cell cycle profile of these cells on Day 7 would be similar to control cells. The authors should at least make the effort to perform FACS analysis (a method that they have used in the manuscript) on Day 5 and Day 7 to clarify this discrepancy.
The discrepancy between their results and the published results begs a concern as to whether an excessive amount of overexpressed TRF1WT in U2OS cells (rather than resolution of persistent telomere cohesion per se) is responsible for causing subtelomere recombination, DNA damage and rapid growth arrest. This concern was not addressed by the authors but needs to be experimentally addressed (e.g. knocking down endogenous TRF1 but not overexpressed TRF1WT in U2OS cells), especially given that the authors have not demonstrated a direct link that resolution of persistent telomere cohesion causes subtelomere recombination.

3. The authors provided their measurement on the frequency of APBs in their rebuttal. They should also cite these APB data and discuss the associated concern in the manuscript.

4. The authors declined to address whether overexpression of TRF1WT causes increased DNA damage, RAD51 foci and growth arrest in BIBR-treated HCT75 PD245 cells. A major concern in this manuscript is about the effect of overexpressed TRF1WT on various phenotypes including DNA damage and growth arrest (see the point #2 above). Therefore, it is critical that the authors address this issue to substantiate their findings.

Reviewer #3 (Remarks to the Author):

The authors have appropriately addressed my comments in their entirety.

Response to Reviewer 2

Reviewer #2 (Remarks to the Author):

The authors have made efforts to address some of the concerns raised previously, however several main issues remain outstanding. This dampens one's enthusiasm to endorse it for publication as it currently stands.

1. The TRF1AA work cited by the authors demonstrates that the tankyrase binding site of TRF1 is responsible for the observed phenotypes (resolution of persistent telomere cohesion, subtelomere recombination) in aged cells overexpressing TRF1WT, but it does not demonstrate the causal relationship between the phenotypes. As a result, the authors have not convincingly shown that there is a causal relationship among phenotypes caused by overexpression of TRF1WT in aged cells. In the absence of experimental evidence for a demonstrated causal relationship between persistent telomere cohesion and subtelomere recombination, their conclusion that persistent telomere cohesion protects against inappropriate recombination is not sufficiently supported.

We have now "toned down" our conclusion "that persistent telomere cohesion protects against inappropriate recombination" throughout the manuscript, as indicated below.

In the abstract in line 8. We changed "The persistent cohesion protects short telomeres from inappropriate recombination." to "Our findings suggest that the persistent cohesion protects short telomeres from inappropriate recombination."

On the bottom of page 8 we already say "suggest" – "Our studies thus far in normal primary cells suggest that telomere shortening that occurs in the absence of telomerase induces persistent telomere cohesion (resulting from the inability of limiting TRF1 to recruit tankyrase 1), which serves as a protective mechanism against subtelomere recombination, DNA damage, and premature activation of senescence."

In the last paragraph of page 10 we already say "suggests"- "The absence of subtelomere recombination upon forced resolution by telomerase further suggests that it is the critically short telomeres that are responsible for the subtelomere recombination."

On page 11 first paragraph we changed "Our data indicate that as telomeres shorten, persistent telomere cohesion driven by insufficient TRF1 (and lack of tankyrase 1 recruitment), protects cells from subtelomere recombination and premature activation of senescence." to "Our data suggest that as telomeres shorten, persistent telomere cohesion driven by insufficient TRF1 (and lack of tankyrase 1 recruitment), protects cells from subtelomere recombination and premature activation of senescence."

On the top of page 12 we changed "Together these data indicate that as telomeres shorten and cells approach senescence, transient persistent telomere cohesion protects cells from subtelomere recombination." to "Together these data suggest that as telomeres shorten and cells approach senescence, transient persistent telomere cohesion protects cells from subtelomere recombination."

In the first paragraph of the discussion on page 12 we changed "As telomeres shorten, the inability to recruit sufficient TRF1 and as a result tankyrase 1 leads to persistent telomere cohesion in mitosis that protects shortened telomeres from inappropriate recombination and DNA damage-induced senescence" to "As telomeres shorten, the inability to recruit sufficient TRF1 and as a result tankyrase 1 leads to persistent telomere cohesion in mitosis, which we suggest, protects shortened telomeres from inappropriate recombination and DNA damage-induced senescence.

On the bottom of page 12 we changed "We showed that persistent cohesion protects cells from illegitimate subtelomere recombination." to "Our data suggest that persistent cohesion protects cells from illegitimate subtelomere recombination."

In the middle of page 13 we changed "Thus, persistent cohesion (in any cell type) likely serves a

protective role by providing shortened (perhaps endogenously damaged) telomeres with a sister for DNA repair and by preventing damage-inducing subtelomere recombination with non-sisters." to "Thus, persistent cohesion (in any cell type) may serve a protective role by providing shortened (perhaps endogenously damaged) telomeres with a sister for DNA repair and by preventing damage-inducing subtelomere recombination with non-sisters."

2. The authors showed that overexpression of TRF1WT in U2OS cells induces DNA damage and cellular senescence, which is in stark contrast to the published report by Silva et al that showed that overexpression of TRF1WT in U2OS does not affect cell cycle profile. The authors attributed this discrepancy to the difference in their ways of doing experiments, e.g. through Day 5 vs Day 7 by Silva et al. It is rather inadequate that the authors just stated that "we cannot say what the FACS analysis would look like at Day 7". Given that their data in Fig. 5O showed that 30% of TRF1WT-overexpressed cells are senesced, it is difficult to imagine that the cell cycle profile of these cells on Day 7 would be similar to control cells. The authors should at least make the effort to perform FACS analysis (a method that they have used in the manuscript) on Day 5 and Day 7 to clarify this discrepancy. The discrepancy between their results and the published results begs a concern as to whether an excessive amount of overexpressed TRF1WT in U2OS cells (rather than resolution of persistent telomere cohesion per se) is responsible for causing subtelomere recombination, DNA damage and rapid growth arrest. This concern was not addressed by the authors but needs to be experimentally addressed (e.g. knocking down endogenous TRF1 but not overexpressed TRF1WT in U2OS cells), especially given that the authors have not demonstrated a direct link that resolution of persistent telomere cohesion causes subtelomere recombination.

In the published report by Silva et al, TRF1 or an empty vector control was overexpressed in U2OS cells by retroviral infection in order to determine if it would influence the FACS profile upon FANCM depletion. Fig. 1h in the paper shows a FACS profile of an empty vector versus TRF1 after 5 days of retroviral infection followed by 2 days of control siRNA transfection. The result shows that the FACS profile between the empty vector and TRF1 is similar. We see no discrepancy between this piece of data and our work. The experiment in Fig. 1h is completely different from what we have done in our paper. We infect U2OS cells with TRF1 lentivirus and immediately (following 48 hours of infection) seed cells at low density for growth curve analysis. We are taking a snapshot at the earliest time possible after infection before cells have a chance to compensate and overgrow. We seed at very low density so that we can monitor growth over time. This is completely different from the experiment in Fig. 1h. In fact, if we allow the cells to sit in the tissue culture dish at higher (more typical) density for several days, the non-senescing cancer cells eventually take over the population. Thus, unlike aged fibroblasts infected with TRF1.WT, the non-senescing U2OS cancer cells eventually continue growing. We now state this in the paper at the bottom of page 9. The result is specific and not due to overexpression because the TRF1.AA allele is expressed to the same extent as WT and has no effect. Reviewer 2 cites this specificity in the first line of point 1 above: *"The TRF1AA work cited by the authors demonstrates that the tankyrase binding site of TRF1 is responsible for the observed phenotypes..".*

3. The authors provided their measurement on the frequency of APBs in their rebuttal. They should also cite these APB data and discuss the associated concern in the manuscript.

On the bottom of page 9 we added "The growth arrest was unlikely due to misregulation of ALT activity, as there was no change in the frequency of ALT-associated PML-bodies (a hallmark of the ALT pathway)."

4. The authors declined to address whether overexpression of TRF1WT causes increased DNA damage, RAD51 foci and growth arrest in BIBR-treated HCT75 PD245 cells. A major concern in this manuscript is about the effect of overexpressed TRF1WT on various phenotypes including DNA damage and growth arrest (see the point #2 above). Therefore, it is critical that the authors address this issue to

substantiate their findings.

We did not decline to address the effect of TRF1.WT in BIBR-treated HTC75 PD245 cells. We pointed out that the purpose of the BIBR-HTC75 experiment was to artificially generate short telomeres and ask if that (in itself) would induce persistent telomere cohesion (in a telomerase positive cancer cell line that did not normally exhibit persistent telomere cohesion) and support subtelomere copying upon TRF1.WT (but not TRF1.AA) overexpression, which it did. We did not see the value in using this artificial system (where telomerase activity is reduced but not eliminated and where cells have been extensively passaged in culture) to perform additional analyses, since we had already shown the consequences of TRF1.WT (versus TRF1.AA) on RAD51, DNA damage, and growth in cells that (naturally) completely lack telomerase (normal aged cells and ALT cancer cells).

Nonetheless, as requested we now provide new data in new Fig. S5F-S5K to address the issue. We infected BIBR-treated HTC75 PD245 cells with Vec, TRF1.WT, and TRF1.AA lentiviruses and performed immunoblot analysis, immunofluorescence analysis for RAD51 and for DNA damage markers γH2AX and 53BP1, and growth curve analysis. Similar to our results in normal aged cells and ALT cancer cells TRF1.WT, but not TRF1.AA, led to an increase in RAD51 foci (Fig. S5F-S5H) and DNA damage foci (Fig. S5I-S5J). We did not observe a growth arrest (Fig. S5K) perhaps due in part to residual telomerase activity in BIBR-treated HTC75 cells. We now state these results at the end of the first paragraph on page 11.

REVIEWERS' COMMENTS:

Reviewer #2 (Remarks to the Author):

The authors showed that overexpression of TRF1WT in aged cells results in several phenotypes (resolution of persistent telomere cohesion, subtelomere recombination, DNA damage and premature senescence). A major concern of this reviewer has been that these phenotypes may represent individual consequences of overexpression of TRF1WT in aged cells rather than causally related phenotypes as claimed by the authors (persistent telomere resolution protects against subtelomere recombination and premature senescence). In this round of the revision, to address this major concern raised in point 1, the authors changed the wording from "indicate" to "suggest" or added the wording "suggest or suggesting" as a way to "tune down" statements regarding the causal relationship. In this reviewer's view, this type of "tune down" has no material impact and does not address the main concern (point 1). In addition, the new experiments they did for point 4 show that overexpression of TRF1 did not induce cellular senescence in aged BIBR-treated HTC76 (PD245) cells, adding to the concern that there is no causal relationship among phenotypes (resolution of persistent telomere cohesion and premature senescence) caused by overexpression of TRF1WT in aged cells. As a result, this reviewer remains unconvinced about their model that persistent telomere cohesion protects aged cells from premature senescence. This reviewer recognizes that two other reviewers were positive and would like to leave the decision to publish the manuscript to the discretion of the Editors.
